# Nanopore-Based Fingerprint Immunoassay Based on Rolling Circle Amplification and DNA Fragmentation

**DOI:** 10.1021/acsnano.2c09889

**Published:** 2023-03-06

**Authors:** Xinqi Kang, Connie Wu, Mohammad Amin Alibakhshi, Xingyan Liu, Luning Yu, David R. Walt, Meni Wanunu

**Affiliations:** ^†^Departments of Bioengineering, ^∇^Physics, and ^‡^Chemistry and Chemical Biology, Northeastern University, Boston, Massachusetts 02115, United States; ¶ Department of Pathology, Brigham and Women’s Hospital, Harvard Medical School and Wyss Institute for Biologically Inspired Engineering at Harvard University, Boston, Massachusetts 02115, United States

**Keywords:** DNA hairpin, α-hemolysin, stable polymer bilayer, biosensing, biomarker, rolling circle amplification

## Abstract

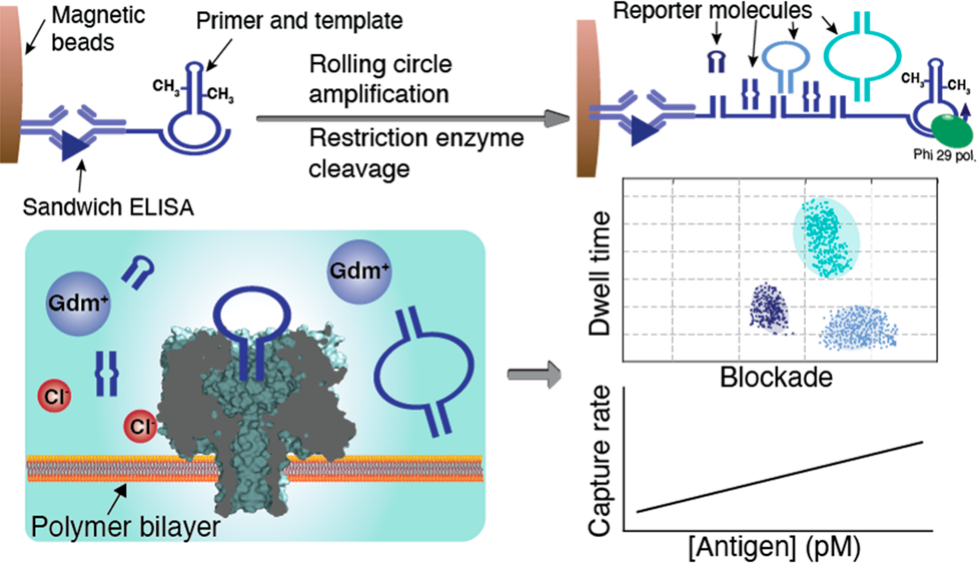

In recent years, nanopore-based sequencers have become robust tools with unique advantages for genomics applications. However, progress toward applying nanopores as highly sensitive, quantitative diagnostic tools has been impeded by several challenges. One major limitation is the insufficient sensitivity of nanopores in detecting disease biomarkers, which are typically present at pM or lower concentrations in biological fluids, while a second limitation is the general absence of unique nanopore signals for different analytes. To bridge this gap, we have developed a strategy for nanopore-based biomarker detection that utilizes immunocapture, isothermal rolling circle amplification, and sequence-specific fragmentation of the product to release multiple DNA reporter molecules for nanopore detection. These DNA fragment reporters produce sets of nanopore signals that form distinctive fingerprints, or clusters. This fingerprint signature therefore allows the identification and quantification of biomarker analytes. As a proof of concept, we quantify human epididymis protein 4 (HE4) at low pM levels in a few hours. Future improvement of this method by integration with a nanopore array and microfluidics-based chemistry can further reduce the limit of detection, allow multiplexed biomarker detection, and further reduce the footprint and cost of existing laboratory and point-of-care devices.

## Introduction

Proteins play important roles in biological processes and thus can serve as valuable biomarkers for various diseases. Detection of many protein-based biomarkers for clinical diagnostics relies on centralized laboratory tests that use enzyme-linked immunosorbent assay (ELISA).^1,2^ These assays have advantages of high sensitivity, high accuracy, and high-throughput sample processing.^2^ As healthcare trends toward patient-centered models, the concept of point-of-care diagnostic devices has become more commonplace. A point-of-care diagnostic device is portable, easy to operate, easily accessible for people living in remote areas, and convenient for monitoring chronic diseases.^1,2^ While there exist hand-held devices that can detect a single analyte, multifunctional immunoassay platforms have generally remained on benchtop formats,^2^ as these technologies are usually based on sandwich ELISA immunocomplex formation which is quantified using fluorescence measurements and therefore require bulky equipment that integrates sample fluidics with cameras, mechanical stages, light sources, and optics. Hence, alternative strategies need to be explored in order to develop point-of-care immunoassay devices.

Over the past three decades, biological nanopores have been developed and evolved into reliable biosensors capable of probing biophysical properties at the single-molecule level, identifying various biomolecules,^3−12^ studying enzyme kinetics,^13−15^ and sequencing DNA and RNA,^16,17^ and currently nanopores are among a handful of candidate tools for single-molecule protein sequencing.^18−20^ The portability of nanopore sensors and the fast measurement times nanopores can deliver position nanopores as ideal choices for point-of-care diagnostic applications. In nanopore sensing, typically a biological porin such as a protein toxin^21−23^ or a synthetic DNA origami pore^24−26^ spontaneously inserts into a thin organic membrane that separates two chambers filled with electrolyte solutions.^27,28^ Applying a voltage across the membrane induces a highly localized electric field across the pore, leading to a steady-state ionic current signal. This electric field draws charged molecules to the nanopore, and as a result, the ionic current is partially occluded. The occlusion time, amplitude of the current blockade, and signal fluctuations can be used to extract the size, charge, and conformation of the molecules. Furthermore, the frequency of each molecular species being captured by nanopores, known as the capture rate, is a function of the molecular concentration. Nevertheless, two prerequisites must be met for general biosensing applications: (i) a signal amplification method must be adopted to enhance the capture rates of rare clinically relevant biomarkers, and (ii) reporter molecules compatible with the nanopore of choice must be developed, as nanopores are very sensitive to size and charge of analytes, which imposes a major limitation on the use of nanopores for sensing proteins with a wide range of molecular weights and charges. The reporter molecules for a given biomarker can be a group of molecules comprising distinct sizes and structures that produce distinct signal patterns (or fingerprints) in the dwell time versus fractional current blockade parameter space.

**Figure 1:**
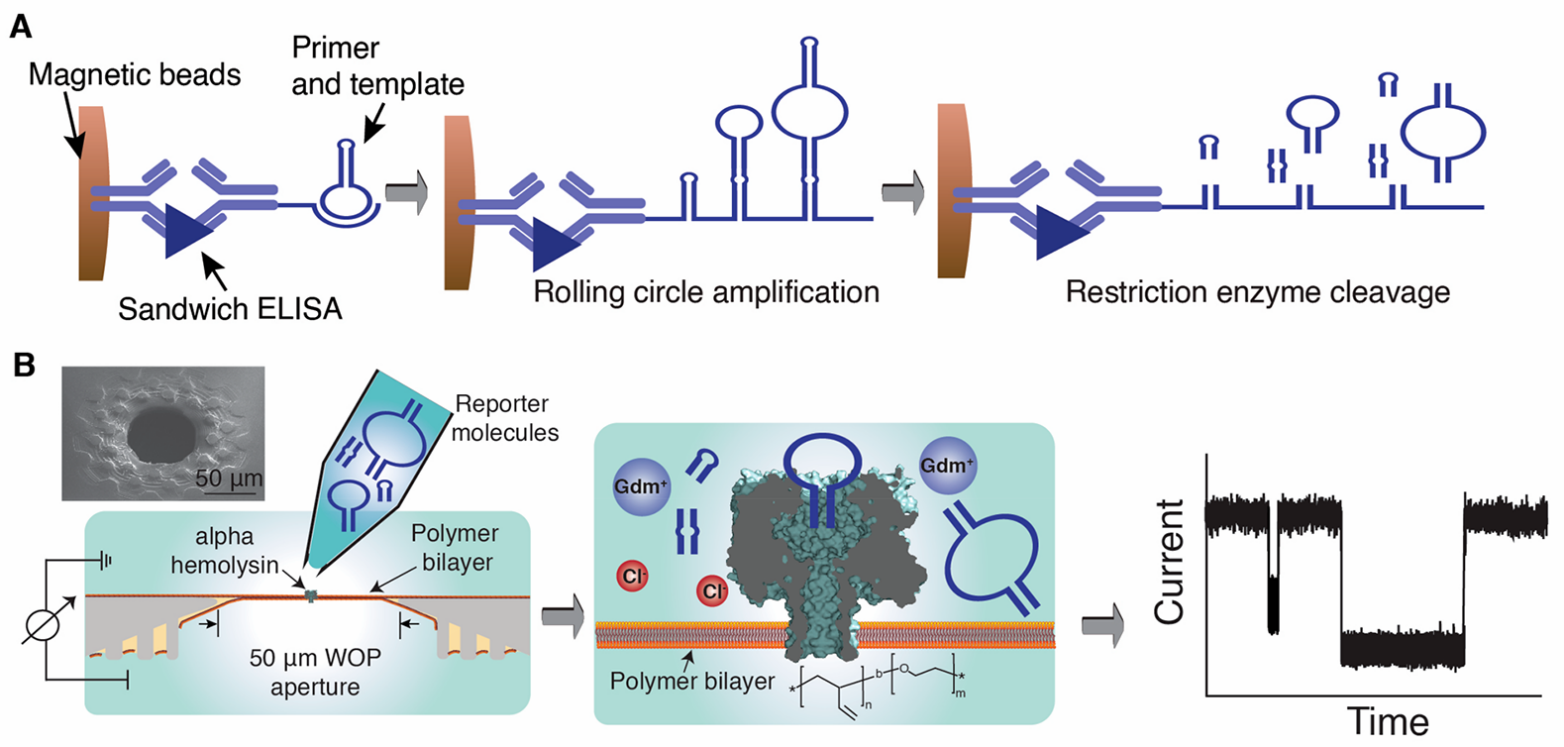
Isothermal rolling circle amplification (RCA) for nanopore-based biomarker quantification. (A) Schematic of RCA-based assay: Capture antibody is conjugated to magnetic beads, and detector antibody is conjugated with DNA primer and template. After the formation of the sandwich immunocomplex, RCA is performed using phi29 polymerase. The circular DNA template contains a DNA hairpin structure that carries a restriction enzyme recognition site. Restriction enzyme-based cleavage produces DNA fragments used as reporter molecules for nanopore-based detection. (B) Schematic of reporter molecule detection using nanopore system. DNA reporter molecules of various sizes generated from panel A were added to the cis chamber of the flow cell. A 50 μm wedge-on-pillar aperture as well as a PBD_11_-PEO_8_ bilayer, which has an α-hemolysin pore inserted, was used for detection of reporter molecules in a guanidinium chloride denaturing environment at high voltage. The inset shows an SEM image of a 50 μm wedge–pillar aperture.

Surrogate reporters for biomarkers such as star-like DNA probes^11^ and ssDNA barcodes^29^ have been previously demonstrated using nanopore sensors. While these studies demonstrate the quantification ability of nanopores, sensing low concentrations remains as a major bottleneck for adopting nanopores as tools for biomarker detection. To fill this gap, here we present an amplification scheme that generates a large number of short DNA molecules as reporter molecules for biomarker quantification which enhances the capture rate of specific molecules and produces events with distinctive fingerprints. In this scheme, we combine isothermal nucleic acid amplification and a sandwich ELISA immunocomplex,^30,31^ with controlled sequence-specific cleavage to enhance the sensitivity of nanopores. Rolling circle amplification (RCA) with the highly processive enzyme phi29 polymerase generates ultralong ssDNA (∼100 knt), an ideal product for signal amplification.^32−34^ We employ this isothermal amplification method that is rapid, cost-effective, easy-to-use, and more tolerant to inhibitory components from crude samples than polymerase chain reaction (PCR), another enzyme-based amplification method.^35^ Restriction enzyme-based digestion of the RCA product generates many DNA fragments of different sizes and structures, which are detected using an α-hemolysin nanopore (Figure 1A). Our measurements reveal an identifiable nanopore fingerprint for a biomarker linked to a circular DNA template. We quantify pM concentrations of a representative protein biomarker, human epididymis protein 4 (HE4), with only 5 min of recording time. We also developed an *in situ* cleavage and amplification (INCA) assay that reduces reaction times and wash steps by using a circular template containing a 5-methylcytosine site that is not amenable to restriction digestion, thereby enabling simultaneous RCA and digestion of the product. Using this approach, we demonstrate the detection of low pM levels of HE4. Our scheme employs reliable and commercially available reagents and circumvents the need for complex DNA nanoparticle synthesis and expensive DNA modifications.

## Results and Discussion

### Reporter Molecule Generation Using RCA

Generally, biosensing with biological nanopore platforms is conducted under low applied bias and at relatively high (nM−μM) analyte concentrations.^21,36^ At 120 mV, DNA hairpin detection was usually conducted at 1 μM concentrations.^21^ While increasing the applied voltage typically increases the molecular capture rate, this advantage is limited by the maximum voltage that can be applied while maintaining a stable membrane. To extend this voltage range, we have previously developed a voltage-stable lipid bilayer platform that allows regularly applied voltages up to 300 mV. With a high applied bias, capture rates increase by as much as 10-fold, thereby reducing detection limits.^28^ However, improving the detection limit and expanding the dynamic range of nanopore sensing to pM levels would vastly increase the utility of nanopore-based sensing of biomarkers. To bridge this gap, we combine immunocapture and RCA of a short circular DNA template to generate a DNA mass that, upon cleavage using restriction enzymes, releases a combination of DNA fragments for nanopore-based detection. Here, a high processivity enzyme, phi29 polymerase, was used to amplify the circular DNA template into a highly repetitive concatemer product. Restriction enzymes, which are known to recognize and cleave DNA at specific sequences, were used to cleave the RCA concatemer product into fragments. We chose the restriction enzymes AluI and RsaI because these two enzymes are active even when cleaving near the end of a DNA duplex. A key element in our design is a DNA hairpin secondary structure within the circular DNA template, which contains a restriction enzyme recognition site that allows isothermal enzymatic digestion (Figure 1A). Upon digestion of the RCA concatemer product, a distinctive collection of DNA fragments is obtained, which produces fingerprint signals during nanopore detection.

We combine here a few of our recent developments in our nanopore sensing platform. First, use of a 50 μm diameter wedge-on-pillar (WOP)^28^ aperture allows high voltage recordings (Figure 1B), which further pushes down the detection limit. Second, use of guanidinium chloride (GdmCl) facilitates passage of the DNA reporter molecules through the pore by compromising the Watson–Crick base-pairing stability. To use GdmCl for detection, we employed the chemically resilient polymer bilayer membrane poly(1,2-butadiene)_11_-*b*-poly(ethylene oxide)_8_ (PBD_11_-PEO_8_) in our platform.^12^

**Figure 2:**
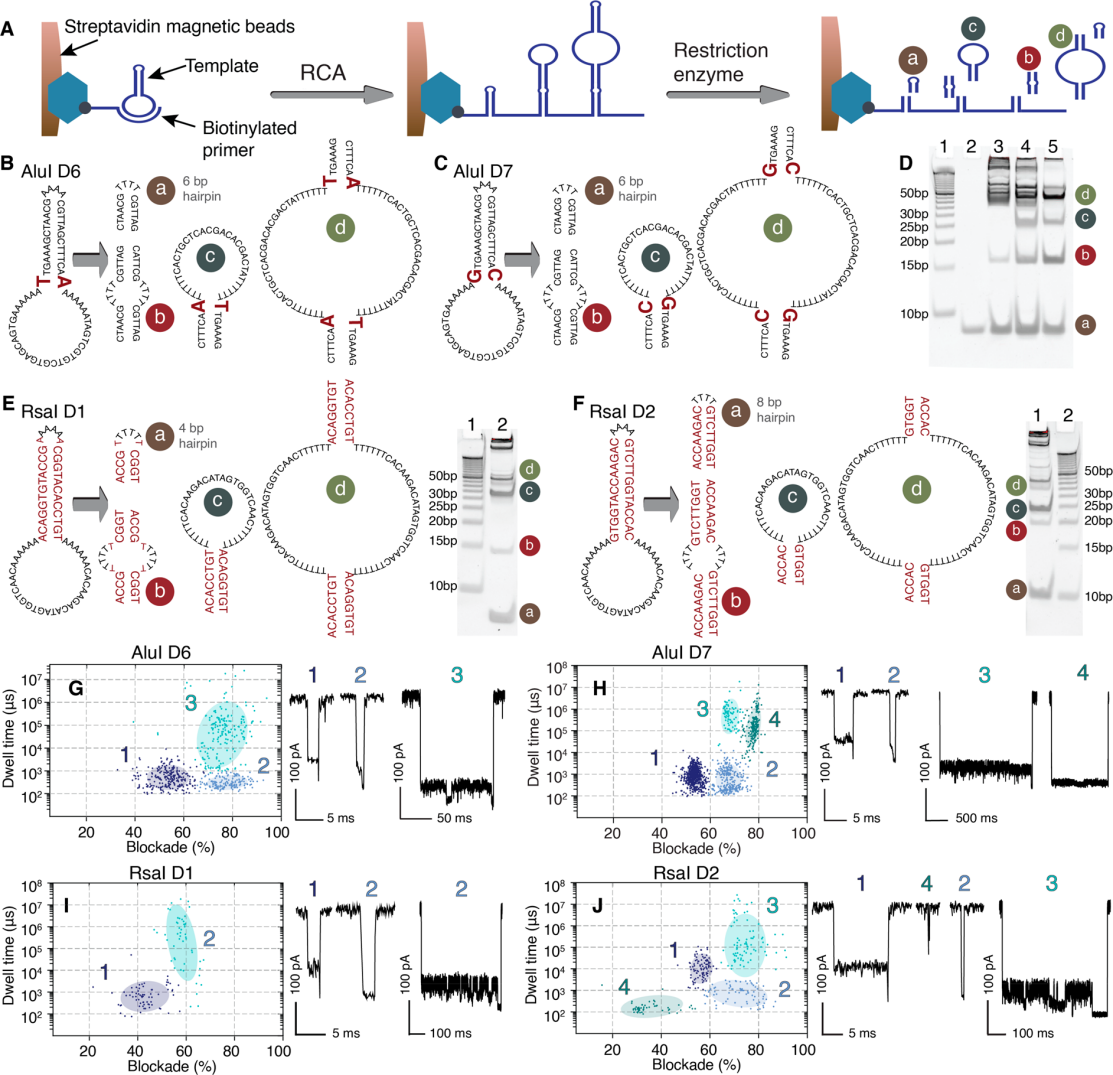
Amplification and sequence-specific cleavage produce nanopore signal fingerprints. (A) Schematic workflow of DNA reporter molecule generation on streptavidin beads. Possible sequences and structures of DNA reporter molecules produced from AluI D6 (B) and AluI D7 (C) and the corresponding 20% native PAGE gel stained by GelRed (D). Lane 1: O’RangeRuler 5 bp DNA Ladder. Lane 2: 6 bp DNA hairpins. Lanes 3 and 4: Reporter molecules generated from circular template AluI D6; lane 3 is AluI cleavage for 30 min, and lane 4 is 60 min. Lane 5: Reporter molecules generated from circular template AluI D7. Possible sequence and structure of DNA reporter molecules produced from RsaI D1 (E) and RsaI D2 (F) and the corresponding 20% native PAGE gel. All restriction digestion was performed at 37 °C for 1 h. Both ladders are O’RangeRuler 5 bp DNA Ladder. Scatter plot of fractional blockade versus dwell time of reporter molecules produced from AluI D6 (G), AluI D7 (H), RsaI D1 (I), and RsaI D2 (J). Bayesian Gaussian mixture model with full covariance type, random state 1, 1 × 10^–9^ convergence threshold, 10,000 initiation number, and random initiation parameters were used for clustering populations in scatter plots. Each cluster is labeled with a number, and the current trace of representative events from each cluster is shown on the right. The concentration of the initial circular DNA template was 10 nM except for AluI D6, which is 2 nM. Experiments were performed in 1 M GdmCl, 1 M KCl, 50 mM Tris, pH 7.6, at 250 mV applied bias. The current signal was lowpass-filtered at 10 kHz.

The primer and templates used in this study are listed in the Supporting Information (Table S1). Successful ligation was confirmed by Exonuclease I and Exonuclease III treatment, since Exonuclease I and Exonuclease digest linear ssDNA and dsDNA while leaving circularized DNA intact (Figure S1). We performed an extensive optimization of the RCA reaction performance as a function of dNTP concentration, phi29 activity, surface (magnetic beads vs ELISA plates), salt type and concentration, magnetic bead concentration, and RCA reaction time (Figure S2). Following optimization, we first characterized the DNA reporter molecules generated from four different primer-template designs on a simple streptavidin–biotin model system (Figure 2A). Since the long ssDNA formed by RCA reaction can hybridize to itself in various ways, there are multiple restriction products that one can obtain. We outline here four different possible DNA fragments, labeled as “a”, “b”, “c”, and “d” (Figure 2A), and whose structures are estimated based on PAGE electrophoresis migration speeds. For both AluI D6 (Figure 2B) and AluI D7 (Figure 2C), the estimated migration speeds for DNA fragments a, b, c, and d correspond to 8, 16, 23, and 46 bp, respectively (gel shown in Figure 2D). The minor appearance of other bands around 50 bp dsDNA is possibly due to the formation of higher-order structures. Similarly, the rough migration speeds of DNA fragments produced from RsaI D1 and RsaI D2 correspond to 7, 14, 24, 48 bp, and 10, 20, 21, 42 bp, respectively (Figure 2E,F). Larger molecular weight bands here are possibly due to incomplete restriction enzyme cleavage, as indicated by a digestion time-course study (Figure 2D, lanes 3 and 4).

Generally, a blunt dsDNA hairpin that is longer than 8 bp is difficult to translocate through an α-hemolysin pore without a denaturing agent (Figure S3),^21,28^ because the free energy of duplex melting is too high. To facilitate this process, we conducted our measurements in a denaturing buffer consisting of 1 M GdmCl, 1 M KCl, and 50 mM Tris, pH 7.6, and used a 250 mV applied bias. Since the product digest consists of several fragments of varying degrees of stability, several types of signals should be obtained. Indeed, we observe multiple populations in the scatter plots of dwell time versus fractional blockade (Figure 2G–J). Employing a Bayesian Gaussian mixture model for clustering these populations, we obtain three clusters of events for AluI D6, and four clusters for AluI D7. We reason that this occurs because AluI cleavage produces two identical 6 bp hairpins; hence, two populations merge into one (Figure 2B,C). Comparing the clustered scatter plots, both AluI D6 and AluI D7 have a cluster “1” centered at ∼55% fractional blockade. We have previously shown that the 6 bp hairpin produces characteristic “shoulder–spike” events with a lower-level blockade around 55% and a sharp deep blockade at the end (Figures S4 and S5).^21^ When examining the single translocation events, this population also has a characteristic two-level blockade pattern (Figure 2G,H, cluster 1). We hypothesize that cluster 1’s in AluI D6 and AluI D7 are translocation events from 6 bp hairpin molecules. The reduction in dwell time is attributed to the action of 1 M GdmCl,^12^ which does not alter the pore structure yet expedites DNA duplex denaturation to facilitate a diffusion-limited turnover of capture events.

Other than the 6 bp hairpin, which is DNA fragment “a”, AluI D6 and AluI D7 can produce fragment “b” that also has 6 bp at both ends, and therefore, it is likely that fragments b and a are in the same cluster. During translocation of molecules a and b, the poly-T_4_ loop is docked on the α-hemolysin pore mouth, since it will adopt a conformation that is not ready to enter the vestibule, and the double-strand stem enters the vestibule.^21^ As the voltage exceeds the energy barrier, the double-strand stem will unzip, and the DNA will translocate the pore as a ssDNA strand forms.^21^ DNA fragments c and d have similar structures but subtle differences in their sequences; that is, AluI D6 has a lower GC content, thus likely corresponding to the broader distribution of clusters 2 and 3 in AluI D6 compared to AluI D7.

Likewise, RsaI D1 and RsaI D2 produce 4 bp and 8 bp hairpins (Figure 2E,F), which also have signature “shoulder-spike” events as shown in our nanopore measurement (Figure 2I,J, cluster 1). The blockade level matches with previous measurements (Figure S5). For reference, we present continuous current traces recorded from reporter molecules generated from four circular templates in Figure S6. Another interesting observation is that when incubating the AluI D6 sample with 1 M GdmCl, 1 M KCl, and 20 mM Tris buffer at 30 °C for 1 h, the nanopore events occur faster, which may be due to the majority of reporter molecules translocating in ssDNA form due to denaturation by GdmCl (Figure S7).

The scatter plots show a distinctive pattern based on the reporter molecules. As an example, we overlaid scatter plots of AluI D7, RsaI D1, and RsaI D2 over AluI D6 (Figure S8A–C). The reproducibility across multiple independent experiments is shown in Figure S8D–I. Thus, by designing the sequence and structure of different RCA templates, we can create characteristic “fingerprints” that have the potential for identifying multiple distinct biomarkers.

### Nanopore Quantification

**Figure 3:**
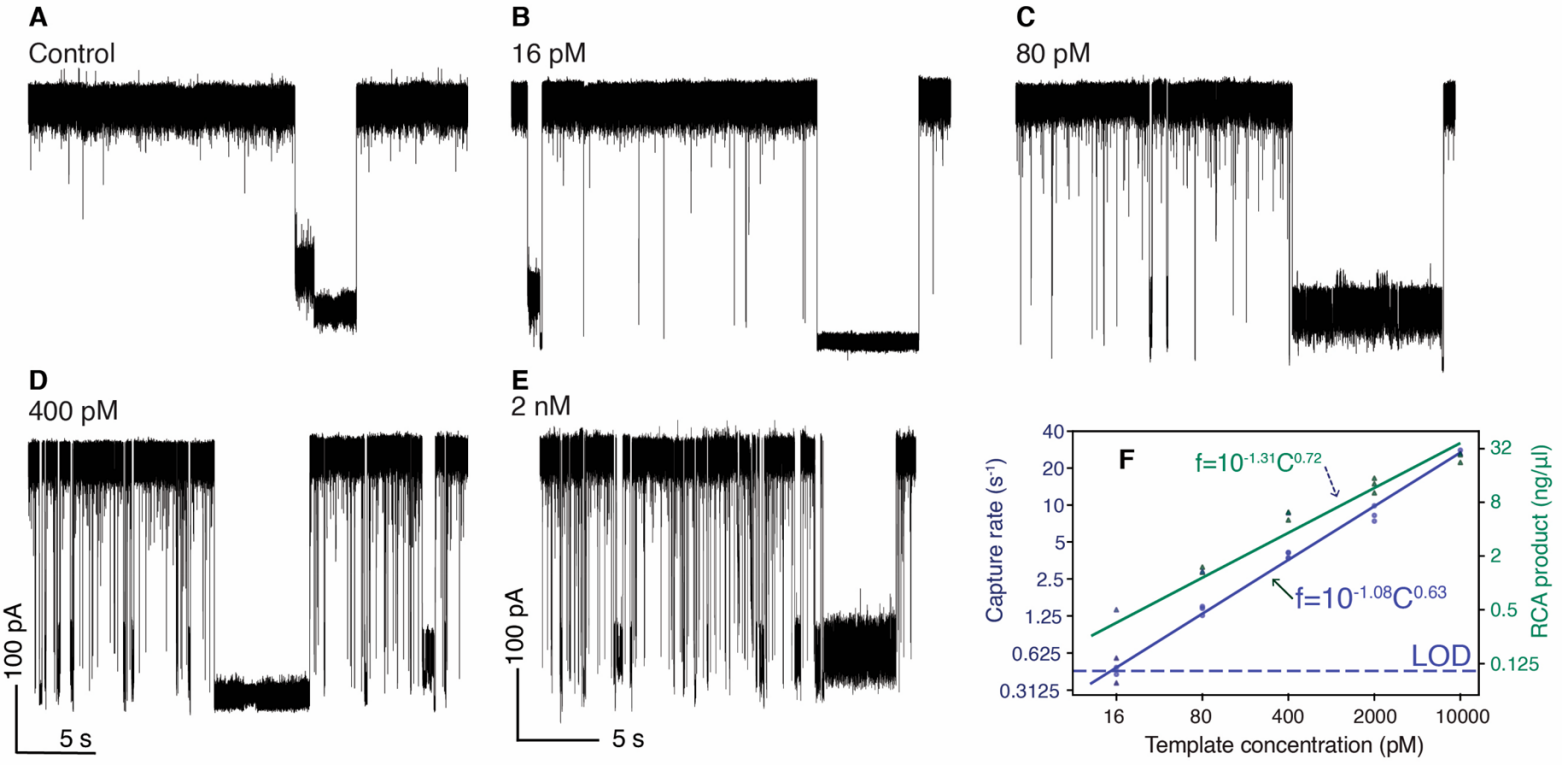
DNA reporter capture rate versus initial circular template concentration. Current traces recorded from no circular template control (A) and a circular DNA template at a concentration of 16 pM (B), 80 pM (C), 400 pM (D), and 2 nM (E). (F) Log–log plot of capture rate (s^–1^) or RCA product mass concentration as a function of initial circular DNA template concentration (pM). The lower limit of detection (LOD) was measured by three standard deviations above the blank. Experiments were performed in 1 M GdmCl, 1 M KCl, 50 mM Tris, pH 7.6, at 250 mV applied bias and lowpass filtered at 10 kHz.

Next, we investigated the limit of detection for reporter molecules amplified from AluI D6 using streptavidin-coated beads and a biotinylated primer as a model system. Figure 3A–E shows representative 20 s current traces recorded at different initial circular template concentrations. At the lowest concentration, a 5 min current recording provided 142 events. Capture rates were obtained by fitting exponential curves^37^ to the distribution of inter-event time. As shown in the log–log plot in Figure 3F, the concentration versus capture rate curve follows a power law with an exponent of 0.63. This exponent is slightly different from the RCA product versus template concentration power exponent of 0.72 (see Figure S2C), the slight difference being possible composition of short versus long DNA fragments in the overall DNA mass, which impacts capture by the nanopore. We also note that for the lowest RCA product concentrations, Qubit quantification results had a large variance, while our nanopore capture rates showed a low variance across multiple experiments. Measured from three blank replicates (no antigen present in sample), the mean capture rate of the blank control was 0.148 s^–1^. Based on the mean value (μ) and standard deviation (σ) of the blank control, an LOD of 14.4 pM is calculated by LOD = 3σ + μ. Importantly, these results show that combining RCA amplification with high voltages enables detection of DNA reporter molecules above 16 pM with a single α-hemolysin pore in a few minutes of detection time. We also compared capture rates between different templates as shown in Figure S9A. The difference in capture rate was similar to the difference in overall RCA product mass, as shown in Figure S2G,H. To demonstrate whether there is a difference in capture rate when using non-AluI D6 primer and template, we tested RsaI D2 at initial template concentrations of 10 and 2 nM, the results of which are shown in Figure S9B.

### INCA Assay and HE4 Quantification

**Figure 4:**
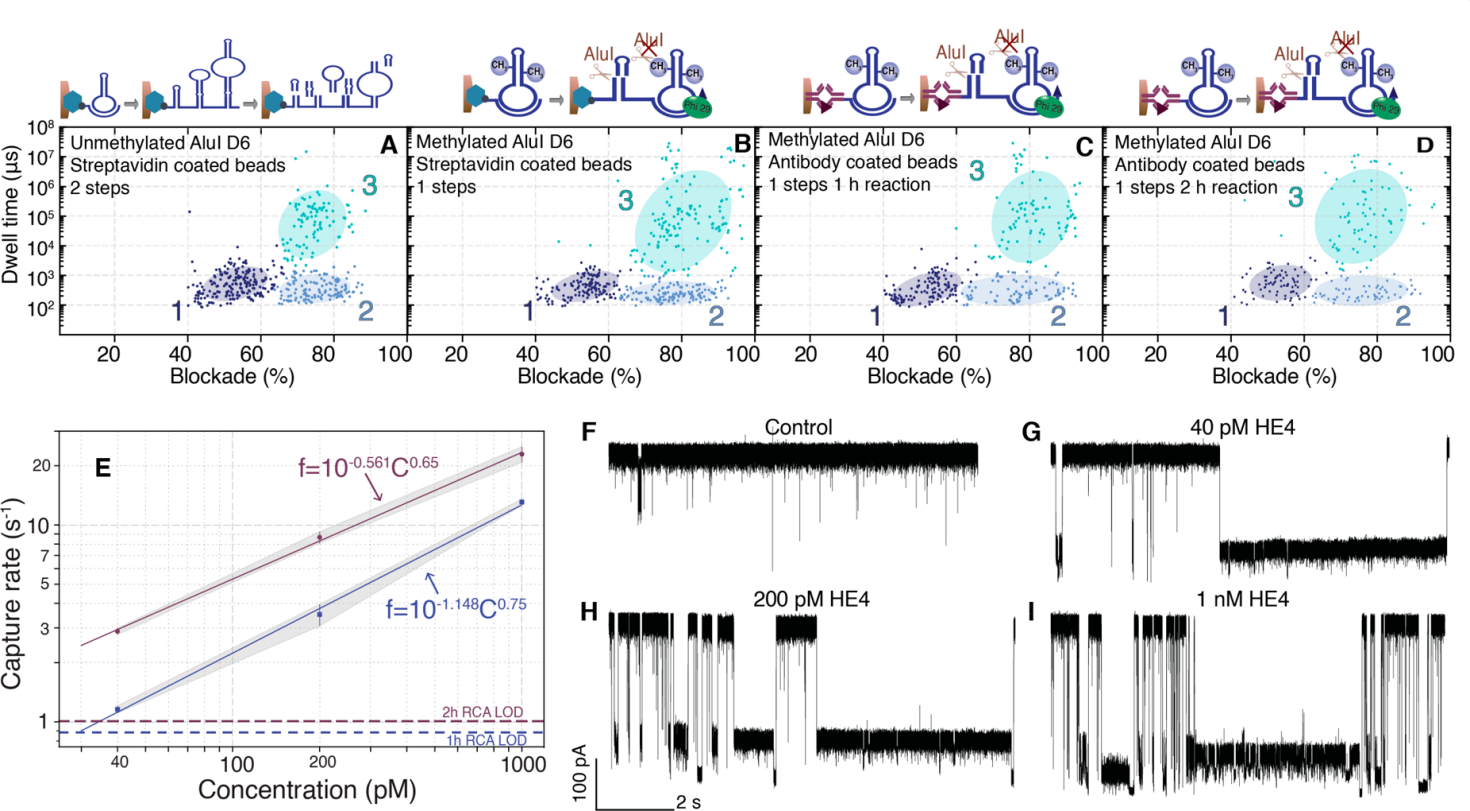
Antibody quantification using methylated circular DNA template. Scatter plot of DNA reporter molecules generated from unmethylated templates (A) and methylated templates (B) immobilized on streptavidin beads. Scatter plots of methylated template immobilized with antibody-conjugated beads and reaction performed for 1 h (C) and 2 h (D). Beads/Stv stands for streptavidin beads and Beads/Ab stands for HE4 antigen conjugated beads. All scatter plots were clustered by Bayesian Gaussian mixture model with full covariance type, random state 1, 1 × 10^–9^ convergence threshold, 10000 initiation number, and random initiation. (E) Capture rate as a function of HE4 concentration. The dashed lines denote assay LODs which were calculated by three standard deviations above the blank. Data were collected from three replicates. Representative current traces were recorded from blank control (F), 40 pM (G), 200 pM (H), and 1 nM HE4 (I). Nanopore measurements were performed in 1 M GdmCl, 1 M KCl, 50 mM Tris, pH 7.6, at 250 mV applied bias. The current signal was lowpass filtered at 10 kHz.

Having successfully demonstrated the rapid detection of low pM concentrations of circular templates using model streptavidin beads, we next applied our platform toward protein biomarker detection. Our first trial in detecting a representative cytokine, interferon-γ, utilized the RsaI D2 template, showing an 8 bp hairpin cluster in the dwell time versus fractional blockade scatter plot. However, as fewer target molecules are expected to be captured by the antibodies compared to the high-affinity streptavidin–biotin system, the capture rate of reporter molecules was significantly lower than that of the streptavidin bead system, at only 0.5 s^–1^ even at 10 nM (Figure S10). We hypothesized that *in situ* cleavage of the RCA product during the amplification reaction can increase reporter molecule yield. To achieve our goal, we exploited the methylation-sensitive property of the AluI restriction enzyme. We created an AluI-resistant circular template methyl-AluI D6 by the addition of two methylated cytosines in the AluI recognition site. Because the incorporated nucleotides are not methylated, the RCA product can be cleaved as phi29 polymerase is acting on the template. The circularization of linear methyl-AluI D6, resistance to AluI digestion, and the successful RCA reaction on the methylated template were confirmed by gel electrophoresis (Figure S11). Phi29 polymerase has a high processivity that could incorporate dNTP ats 2280 nt/min at 30 °C.^38^ In contrast, restriction enzyme cleavage speeds are generally slow. To make sure cleavage is suitable for detection, we varied a few different reaction parameters such as dNTP concentration, phi29 polymerase concentration, and AluI enzyme concentration, and the results were not very different within the range of concentration tested (Figure S12). The scatter plots of DNA reporter molecules generated from unmethylated and methylated AluI D6, both captured on streptavidin beads at an initial template concentration of 400 pM, are shown in Figure 4A,B. Clustering with a Bayesian Gaussian mixture model shows three populations, with the 6 bp hairpin denoted as population 1. The corresponding trace is shown in Figure S13A,B. Consistent with our hypothesis that *in situ* cleavage of the RCA product would increase reporter molecule yield, the methylated template improved the nanopore capture rate by nearly 4-fold over the unmethylated template (13.07 versus 3.43 s^–1^). Next, we applied the INCA platform to the detection of the protein biomarker HE4, using capture antibody-conjugated magnetic beads and a detector antibody conjugated to the annealed primer and methylated template pair. Consistent with our results, nanopore data with methylated template and antibody conjugated beads show similar population clusters as with streptavidin beads (Figure 4C,D). The overlaid scatter plots are shown in Figure S13C–E. Here we note that population 3 from the reporter molecules generated from the methylated template demonstrates larger blockades and long dwell time events, possibly due to a relatively large amount of larger reporter DNA fragment present in the mixture.

We measured the concentration dependence on capture rates for both 1 and 2 h reaction times and found that 2 h RCA reaction times show an approximate 1.7-fold higher capture rates than 1 h reaction times (Figure 4E). The LODs for a 1 h and a 2 h reaction are 28.8 pM and 7.4 pM, respectively. The current traces for 2 and 1 h reaction times at various HE4 concentrations are shown in Figures 4F–I and S14, respectively. The INCA assay not only increases the yield of reporter molecules but also reduces the need for washing steps between RCA and restriction enzyme treatment.

**Figure 5:**
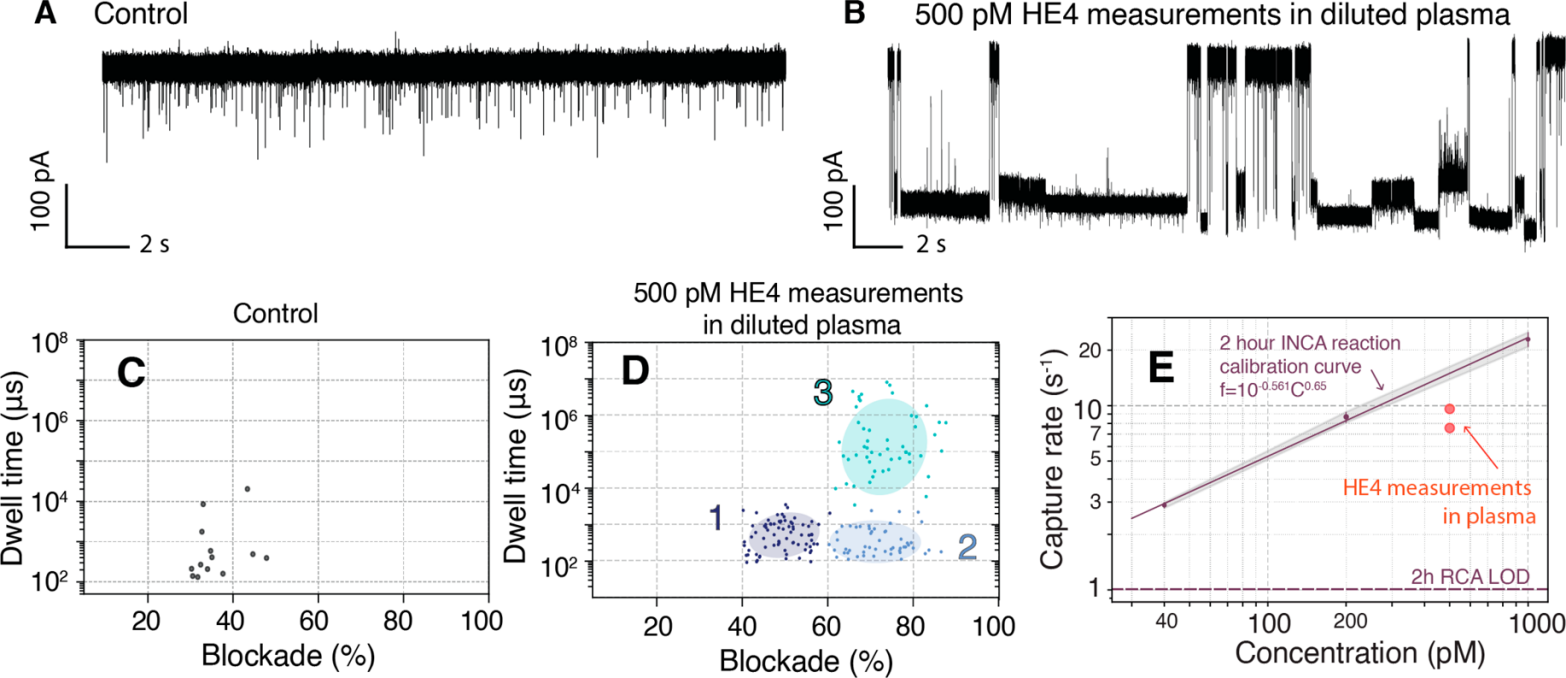
HE4 concentration measurements in human plasma using INCA. Representative current traces recorded from blank control (A) and 500 pM HE4 in 4-fold diluted plasma samples (B) and corresponding scatter plots (C and D). The INCA reaction was performed for 2 h. Bayesian Gaussian mixture model with full covariance type, random state 1, 1 × 10^–9^ convergence threshold, 10000 initiation number, and random initiation was used for clustering. (E) Capture rate measurement of corresponding HE4 sample plotted on the calibration curve. LOD was measured by three standard deviations above the blank. Experiments were performed in 1 M GdmCl, 1 M KCl, 50 mM Tris, pH 7.6, at 250 mV applied bias. The current signal was lowpass filtered at 10 kHz.

Finally, we explored the INCA performance in biological fluids. As a proof of concept, we measured HE4 in 4-fold diluted human serum. The representative current traces and corresponding scatterplots of the blank control and 500 pM HE4 are shown in Figure 5A–D. Clustering with a Bayesian Gaussian mixture model shows consistent results as discussed above. The calculated capture rate at 500 pM HE4 in serum was 8.58 s^–1^, which correspond to 199 nM. The recovery is 37.6%. The low recoveries could be attributed to matrix effects from interfering components in biological fluids. This issue could be addressed by multiple strategies, such as further dilutions, addition of blocking agents/detergents, etc.

## Conclusions

We have developed a robust biological nanopore platform for biomarker detection based on isothermal amplification of DNA reporters followed by enzymatic DNA fragmentation. Our signal amplification assay bridges the gap between biological nanopores and point-of-care biomarker quantification by allowing rapid biomarker detection at pM levels. As a proof of concept, we demonstrate its capability of quantifying a representative protein biomarker, HE4, with 7.4 pM LOD.

One of the advantages of RCA as a signal amplification method is its high adaptability toward a wide range of targets, including proteins, mRNA, and single nucleotide polymorphisms (SNPs).^35,39^ For example, with the addition of T4 ligase and padlock probes, the assay can be modified for SNP detection. In addition, compared to other isothermal amplification methods, RCA can be conducted at relatively low temperatures and does not require fine thermal control, thus facilitating integration into a portable and simple device.^35^ However, to create a distinct spectrum, we can also employ loop-mediated isothermal amplification, which can potentially provide higher sensitivities and a wider dynamic range. As shown in Figure S15, after the formation of a sandwich immunocomplex, heat-mediated release of aptamer molecules, and loop-mediated isothermal amplification, long dsDNA products are generated that can be digested by restriction enzymes into DNA fragments similar to the reporter molecules employed in this article. Thus, our main idea of a nanopore spectrum is highly versatile and can be applied to other nucleic acid amplification methods.

Further improvements can be made to further expand this method and improve detection limits. Enhancing the capture rates of DNA fragments by shifting from wild-type α-hemolysin to an electroosmotically enhanced α-hemolysin mutant,^40^ applying salt gradients across the pore,^41^ scaling up to an array of pores to integrate the detection events in similar sample volumes, and further reducing the sample volume via an automated microfluidic system can potentially improve analytical sensitivity to femtomolar or potentially attomolar levels. Further work will focus on achieving even lower LODs, faster reaction times, and multiplexing, which are desirable for many clinical applications, especially in biological fluids such as saliva that can be noninvasively collected but contain much lower biomarker concentrations.

## Methods

### SU-8 Wedge–Pillar Aperture Fabrication

The SU-8 aperture was fabricated on a 500 μm thick ⟨100⟩ Si wafer with 200 μm silicon dioxide and a 50 nm thick silicon nitride layer coated on both sides. The 200 μm silicon dioxide buried underneath the silicon nitride serves to reduce the capacitive noise of the chips. The wafer was the first pattern with an array of 1 mm squares using the standard photolithography method. Then the wafer was etched by 150 W 1 min of SF_6_ reactive ion etching, 50 min of buffered oxide etch (BOE). Next, on the backside, the wafer was spun coated with 25 μm thick SU-8 3025, soft baked at 95 °C, constant power 275 W for 12.5 s, post exposure baked at 95 °C for 4 min 30 s, and developed for 6 min. Greyscale photolithography was used to create wedge–pillars as we discussed in previous work. Finally, the 500 μm Si layer, silicon dioxide layer, and silicon nitride layer were removed using standard KOH, BOE, SF6 reactive ion etching while a single side etcher was used to protect the SU-8 wedge-on-pillar aperture on the backside.

### Polymer Bilayer Painting and Nanopore Measurement

First, the wedge–pillars aperture was pretreated with 1 μL of hexane dissolved PBD_11_-PEO_8_ (Catalog #P41807C-BdEO; PolymerSource, Montreal, Quebec, Canada) (5 mg/mL) on each side. After the hexane evaporated, the chip was mounted on our customized flow cell. The trans chamber was filled with 1 M GdmCl, 1 M KCl, 50 mM Tris, pH 7.6 electrolyte, and the cis chamber was filled 50 μL of reporter molecules sample plus 150 μL of 1.33 M GdmCl, 1.33 M KCl, 66.5 mM Tris pH 7.6 so that the final electrolyte concentration is the same as the trans. A Ag/AgCl pair was inserted into the electrolyte and connected to the patch amplifier (Axopatch 200B; Molecular Devices, San Jose, CA). Decane-dissolved PBD_11_-PEO_8_ (20 mg/mL) was painted across the aperture using a pipet. After confirmation of bilayer formation by checking the capacitance, 0.5 μL of 25 μg/mL α-hemolysin was added to the cis chamber until single-channel insertion was observed. Current signals were collected at 250 kHz sampling rates, lowpass filtered to 10 kHz, and analyzed using Pyth-Ion. For this analysis, the threshold value for the current to detect events was 30% of the open pore current value.

### Rolling Circle Amplification Assay on Streptavidin Beads

Phi29 polymerase was purchased from Enzymatics (P7020-HC-L); restriction enzymes AluI (R0137S), RsaI (R0167S), ExoI (M0293S), ExoIII (M0206S), and dNTPs (N0447L) were purchased from New England Biolabs (NEB); CircLigase I was purchased from Lucigen (CL4111K); and all oligonucleotides were purchased from Integrated DNA Technologies (IDT).

The linear template ligation conditions were 500 nM single-stranded linear DNA, 50 mM MOPS, 10 mM KCl, 5 mM MgCl_2_, 2.5 mM MnCl_2_, 50 μM ATP, 1 mM DTT, 5 U/μL CircLigase I, pH 7.5, 60 °C for 10 h ,and 80 °C for 10 min for inactivation. Later, the circular templates were mixed with primer at a 1:1 ratio and incubated at 37 °C for 15 min.

A suspension of streptavidin magnetic beads (Dynabeads M-270 Streptavidin, Invitrogen) was transferred to an autoclaved PCR tube and washed with 2× Binding&Washing buffer (2 M NaCl, 1 mM EDTA,10 mM Tris-HCl, pH 7.5) 3 times. The biotinylated primer-template hybrid was bound at the condition of 50 μL of the final concentration of 1 μg/μL streptavidin magnetic beads, 1 M NaCl, 0.5 mM EDTA,5 mM Tris-HCl, pH 7.5, 25 °C for 30 min with gently shaking with tube rotator (Roto-Therm). After DNA binding, beads were washed 3 times with 50 μL of 1× Binding&Washing buffer, moved to a new autoclaved PCR tube, and washed 2 times with 50 μL of 1× phi29 reaction buffer (100 mM (NH_4_)_2_SO_4_, 100 mM MgCl_2_, 500 mM Tris-HCl, 40 mM DTT, pH 7.5). The reaction volume is 50 μL. Generally, the reaction was carried out in 1 mM dNTPs, 2 U/μL phi29 polymerase, 1 μg/μL 2.8 μm magnetic beads, 10 mM (NH_4_)_2_SO_4_, 10 mM MgCl_2_, 50 mM Tris-HCl, 4 mM DTT, 0.05% Tween-20, pH 7.5 with gentle mixing at 37 °C for 1 h unless indicated otherwise. The reaction was stopped by the addition of EDTA to a final concentration of 50 mM.

For characterizing RCA reaction yield, the samples were washed 5 times with Binding&Washing buffer and incubated at 90 °C for 30 min in 50 uL of 95% formamide, 10 mM EDTA, pH 8.2. After the sample was cooled down, Qubit ssDNA dye (Invitrogen Q10212) was used and later quantified by Qubit Fluorometer. 0.3% agarose gel was run at 50 V at 4 °C for 1 h and later stained by Gelred.

For producing reporter molecules, the samples were washed 5 times with 1× NEB4 buffer (50 mM potassium acetate, 20 mM Tris-acetate, 10 mM magnesium acetate, 1 mM DTT, pH 7.9). The restriction enzyme cleavage experiments were performed in 50 μL reaction volume, 1× NEB4 buffer, 20 U restriction enzyme, with gentle mixing at 37 °C for 1 h. In situ cleave and amplification (INCA) was performed in 1 mM dNTPs, 2 U/μL phi29 polymerase, 0.4 U/μL restriction enzyme, 1 μg/μL 2.8 μm magnetic beads, 50 mM Potassium Acetate, 20 mM Tris-acetate, 10 mM magnesium acetate, 4 mM DTT, 0.05% Tween-20, pH 7.9, and 50 μL reaction volume. After enzyme digestion, magnetic beads were separated, and the 50 μL of supernatants were mixed with 150 μL of 1.33 M GdmCl, 1.33 M KCl, 66.5 mM Tris pH 7.6 so that final GdmCl and KCl concentrations are 1 M. The reporter molecules were characterized by 20% native PAGE which are run at 160 V for 2 h, stained with Gelred, and visualized with a Biorad PharosFX imaging system.

For the streptavidin plates experiment, the same RCA reaction conditions were used except the reaction volume is 100 μL and the biotinylated primer binding is 1 h instead of 30 min. The plate was purchased from Thermofisher (Nunc immobilizer streptavidin).

### Capture Antibody Conjugation to Beads for HE4 Assay

For conjugation, 4.2 × 10^8^ paramagnetic carboxylated beads (Homebrew Singleplex beads, Quanterix) were washed three times with 300 μL of Bead Wash Buffer (Quanterix) and two times with 300 μL of cold Bead Conjugation Buffer (Quanterix) before resuspending in 291 μL of cold Bead Conjugation Buffer. The carboxyl groups on the beads were activated by adding 9 μL of freshly dissolved 1-ethyl-3-(3-(dimethylamino)propyl) carbodiimide hydrochloride (EDC) and shaken at 4 °C for 30 min. The beads were then washed once with 300 μL of cold Bead Conjugation Buffer and resuspended in 300 μL of 0.167 mg/mL capture antibody (MAB62741, R&D Systems) in cold Bead Conjugation Buffer. Antibody conjugation was carried out by shaking the beads at 4 °C for 2 h. The beads were then washed twice with 300 μL of Bead Wash Buffer before resuspending in 300 μL of Bead Blocking Buffer (Quanterix) and shaking at room temperature for 30 min. After blocking, the beads were washed with 300 μL of Bead Wash Buffer and 300 μL of Bead Diluent (Quanterix) and resuspended in Bead Diluent for storage at 4 °C. The beads were counted with a Beckman Coulter Z1 Particle Counter.

### Detector Antibody Conjugation

The primer–template hybrid for conjugation to detector antibody was prepared by first annealing a 5′ azide-modified primer (36.1 μM) and a linear methylated AluI D6 template (37.9 μM) in NEBNext Quick Ligation Buffer (New England Biolabs). The mixture was heated at 95 °C for 2 min and allowed to slowly cool to room temperature over 1.5 h. The template was then ligated by adding T4 DNA ligase and incubating at room temperature for 3 h. After ligation, a 7K MWCO Zeba spin desalting column (Thermo Fisher Scientific) was used to buffer exchange the primer–template pair into phosphate buffered saline (PBS) with 1 mM EDTA. The detector antibody (AF6274, R&D Systems) was reconstituted to 1 mg/mL in PBS and incubated with a 20-fold molar excess of dibenzocyclooctyne-PEG4-*N*-hydroxysuccinimidyl ester (DBCO-PEG4-NHS, MilliporeSigma) at room temperature for 30 min before purification with a 10K Amicon Ultra-0.5 mL centrifugal filter in PBS with 1 mM EDTA. The DBCO-modified detector antibody was then mixed with a 2-fold molar excess of the ligated primer-template and incubated at 4 °C overnight. The antibody–DNA conjugate was aliquoted and stored at −80 °C in PBS with 5 mM EDTA, 0.1% BSA, and 0.02% sodium azide.

### HE4 INCA Assay

Immunoassays were performed in 96-well plates (Greiner Bio-One, 655096). For each sample to be measured, 1 × 10^6^ beads diluted in 10 μL of Homebrew Sample Diluent (Quanterix) were added to 100 μL of sample diluted in Homebrew Sample Diluent. The plate was sealed and shaken at room temperature for 1 h before washing three times with System Wash Buffer 1 (Quanterix) using a BioTek 405 TS Microplate Washer. The beads were then resuspended in 100 μL of detector antibody-DNA conjugate (0.6 μg/mL diluted in Homebrew Sample Diluent) and shaken at room temperature for 15 min. After washing 10 times with System Wash Buffer 1, the beads were transferred to a new 96-well plate and resuspended in 60 μL of RCA reaction mix consisting of 1 U/μL phi29, 0.2 U/μL AluI, 1 mM dNTP, 0.05% Tween-20, and 1× NEBuffer 4. The plate was shaken at 37 °C for 1.5 h, and the RCA reaction was quenched by adding 6 μL of 500 mM EDTA before nanopore measurements of the supernatant.

## Supporting Information Available

Template ligation, optimization of rolling circle amplification, nanopore measurement of pure DNA hairpins, nanopore current traces, reproducible clusters across multiple independent experiments, and ligation of methylated templates and its optimization (PDF)

## References

[ref1] Vashist S. K. Point-of-Care Diagnostics: Recent Advances and Trends. Biosensors (Basel) 2017, 7 (4), 62 10.3390/bios7040062. 29258285 PMC5746785

[ref2] St John A.; Price C. P. Existing and Emerging Technologies for Point-of-Care Testing. Clin Biochem Rev. 2014, 35 (3), 155–167. 25336761 PMC4204237

[ref3] Aw J. G. A.; Lim S. W.; Wang J. X.; Lambert F. R. P.; Tan W. T.; Shen Y.; Zhang Y.; Kaewsapsak P.; Li C.; Ng S. B.; et al. Determination of isoform-specific RNA structure with nanopore long reads. Nat. Biotechnol. 2021, 39 (3), 336–346. 10.1038/s41587-020-0712-z. 33106685

[ref4] Wang J.; Li M. Y.; Yang J.; Wang Y. Q.; Wu X. Y.; Huang J.; Ying Y. L.; Long Y. T. Direct Quantification of Damaged Nucleotides in Oligonucleotides Using an Aerolysin Single Molecule Interface. ACS Cent Sci. 2020, 6 (1), 76–82. 10.1021/acscentsci.9b01129. 31989027 PMC6978832

[ref5] Wang Y.; Zheng D.; Tan Q.; Wang M. X.; Gu L.-Q. Nanopore-based detection of circulating microRNAs in lung cancer patients. Nat. Nanotechnol. 2011, 6 (10), 668–674. 10.1038/nnano.2011.147. 21892163 PMC3189330

[ref6] Restrepo-Perez L.; Huang G.; Bohlander P. R.; Worp N.; Eelkema R.; Maglia G.; Joo C.; Dekker C. Resolving Chemical Modifications to a Single Amino Acid within a Peptide Using a Biological Nanopore. ACS Nano 2019, 13 (12), 13668–13676. 10.1021/acsnano.9b05156. 31536327 PMC6933820

[ref7] Ji Z.; Guo P. Channel from bacterial virus T7 DNA packaging motor for the differentiation of peptides composed of a mixture of acidic and basic amino acids. Biomaterials 2019, 214, 119222 10.1016/j.biomaterials.2019.119222. 31158604 PMC6724551

[ref8] Ji Z.; Kang X.; Wang S.; Guo P. Nano-channel of viral DNA packaging motor as single pore to differentiate peptides with single amino acid difference. Biomaterials 2018, 182, 227–233. 10.1016/j.biomaterials.2018.08.005. 30138785 PMC6309972

[ref9] Ouldali H.; Sarthak K.; Ensslen T.; Piguet F.; Manivet P.; Pelta J.; Behrends J. C.; Aksimentiev A.; Oukhaled A. Electrical recognition of the twenty proteinogenic amino acids using an aerolysin nanopore. Nat. Biotechnol. 2020, 38 (2), 176–181. 10.1038/s41587-019-0345-2. 31844293 PMC7008938

[ref10] Chuah K.; Wu Y.; Vivekchand S. R. C.; Gaus K.; Reece P. J.; Micolich A. P.; Gooding J. J. Nanopore blockade sensors for ultrasensitive detection of proteins in complex biological samples. Nat. Commun. 2019, 10 (1), 2109 10.1038/s41467-019-10147-7. 31068594 PMC6506515

[ref11] He L.; Tessier D. R.; Briggs K.; Tsangaris M.; Charron M.; McConnell E. M.; Lomovtsev D.; Tabard-Cossa V. Digital immunoassay for biomarker concentration quantification using solid-state nanopores. Nat. Commun. 2021, 12 (1), 5348 10.1038/s41467-021-25566-8. 34504071 PMC8429538

[ref12] Yu L.; Kang X.; Alibakhshi M. A.; Pavlenok M.; Niederweis M.; Wanunu M. Stable polymer bilayers for protein channel recordings at high guanidinium chloride concentrations. Biophys. J. 2021, 120 (9), 1537–1541. 10.1016/j.bpj.2021.02.019. 33617833 PMC8204206

[ref13] Meng F. N.; Ying Y. L.; Yang J.; Long Y. T. A Wild-Type Nanopore Sensor for Protein Kinase Activity. Anal. Chem. 2019, 91 (15), 9910–9915. 10.1021/acs.analchem.9b01570. 31241901

[ref14] Lieberman K. R.; Dahl J. M.; Mai A. H.; Cox A.; Akeson M.; Wang H. Kinetic mechanism of translocation and dNTP binding in individual DNA polymerase complexes. J. Am. Chem. Soc. 2013, 135 (24), 9149–9155. 10.1021/ja403640b. 23705688 PMC3738007

[ref15] Harrington L.; Alexander L. T.; Knapp S.; Bayley H. Single-Molecule Protein Phosphorylation and Dephosphorylation by Nanopore Enzymology. ACS Nano 2019, 13 (1), 633–641. 10.1021/acsnano.8b07697. 30588793

[ref16] Noakes M. T.; Brinkerhoff H.; Laszlo A. H.; Derrington I. M.; Langford K. W.; Mount J. W.; Bowman J. L.; Baker K. S.; Doering K. M.; Tickman B. I.; et al. Increasing the accuracy of nanopore DNA sequencing using a time-varying cross membrane voltage. Nat. Biotechnol. 2019, 37 (6), 651–656. 10.1038/s41587-019-0096-0. 31011178 PMC6658736

[ref17] Lu H.; Giordano F.; Ning Z. Oxford Nanopore MinION Sequencing and Genome Assembly. Genomics Proteomics Bioinformatics 2016, 14 (5), 265–279. 10.1016/j.gpb.2016.05.004. 27646134 PMC5093776

[ref18] Wanunu M. Back and forth with nanopore peptide sequencing. Nat. Biotechnol. 2022, 40 (2), 172–173. 10.1038/s41587-021-01205-x. 35058623

[ref19] Yan S.; Zhang J.; Wang Y.; Guo W.; Zhang S.; Liu Y.; Cao J.; Wang Y.; Wang L.; Ma F.; et al. Single Molecule Ratcheting Motion of Peptides in a Mycobacterium smegmatis Porin A (MspA) Nanopore. Nano Lett. 2021, 21 (15), 6703–6710. 10.1021/acs.nanolett.1c02371. 34319744

[ref20] Alfaro J. A.; Bohlander P.; Dai M.; Filius M.; Howard C. J.; van Kooten X. F.; Ohayon S.; Pomorski A.; Schmid S.; Aksimentiev A.; et al. The emerging landscape of single-molecule protein sequencing technologies. Nat. Methods 2021, 18 (6), 604–617. 10.1038/s41592-021-01143-1. 34099939 PMC8223677

[ref21] Vercoutere W.; Winters-Hilt S.; Olsen H.; Deamer D.; Haussler D.; Akeson M. Rapid discrimination among individual DNA hairpin molecules at single-nucleotide resolution using an ion channel. Nat. Biotechnol. 2001, 19 (3), 248–252. 10.1038/85696. 11231558

[ref22] Fahie M. A.; Liang L.; Avelino A. R.; Pham B.; Limpikirati P.; Vachet R. W.; Chen M. Disruption of the open conductance in the beta-tongue mutants of Cytolysin A. Sci. Rep 2018, 8 (1), 3796 10.1038/s41598-018-22009-1. 29491391 PMC5830503

[ref23] Liu A.; Zhao Q.; Krishantha D. M.; Guan X. Unzipping of Double-stranded DNA in Engineered α-Hemolysin Pores. J. Phys. Chem. Lett. 2011, 2 (12), 1372–1376. 10.1021/jz200525v. 21709813 PMC3119559

[ref24] Hernandez-Ainsa S.; Keyser U. F. DNA origami nanopores: developments, challenges and perspectives. Nanoscale 2014, 6 (23), 14121–14132. 10.1039/C4NR04094E. 25325422

[ref25] Gopfrich K.; Li C. Y.; Ricci M.; Bhamidimarri S. P.; Yoo J.; Gyenes B.; Ohmann A.; Winterhalter M.; Aksimentiev A.; Keyser U. F. Large-Conductance Transmembrane Porin Made from DNA Origami. ACS Nano 2016, 10 (9), 8207–8214. 10.1021/acsnano.6b03759. 27504755 PMC5043419

[ref26] Ishikawa D.; Suzuki Y.; Kurokawa C.; Ohara M.; Tsuchiya M.; Morita M.; Yanagisawa M.; Endo M.; Kawano R.; Takinoue M. DNA origami nanoplate-based emulsion with designed nanopore function. Angew. Chem., Int. Ed. Engl. 2019, 58 (43), 15299–15303. 10.1002/anie.201908392. 31411794

[ref27] Morton D.; Mortezaei S.; Yemenicioglu S.; Isaacman M. J.; Nova I. C.; Gundlach J. H.; Theogarajan L. Tailored Polymeric Membranes for Mycobacterium Smegmatis Porin A (MspA) Based Biosensors. J. Mater. Chem. B 2015, 3 (25), 5080–5086. 10.1039/C5TB00383K. 26413301 PMC4582436

[ref28] Kang X.; Alibakhshi M. A.; Wanunu M. One-Pot Species Release and Nanopore Detection in a Voltage-Stable Lipid Bilayer Platform. Nano Lett. 2019, 19 (12), 9145–9153. 10.1021/acs.nanolett.9b04446. 31724865

[ref29] Liu L.; Li T.; Zhang S.; Song P.; Guo B.; Zhao Y.; Wu H. C. Simultaneous Quantification of Multiple Cancer Biomarkers in Blood Samples through DNA-Assisted Nanopore Sensing. Angew. Chem., Int. Ed. Engl. 2018, 57 (37), 11882–11887. 10.1002/anie.201803324. 29697902

[ref30] Wu C.; Dougan T. J.; Walt D. R. High-Throughput, High-Multiplex Digital Protein Detection with Attomolar Sensitivity. ACS Nano 2022, 16 (1), 1025–1035. 10.1021/acsnano.1c08675. 35029381 PMC9499451

[ref31] Wu C.; Garden P. M.; Walt D. R. Ultrasensitive Detection of Attomolar Protein Concentrations by Dropcast Single Molecule Assays. J. Am. Chem. Soc. 2020, 142 (28), 12314–12323. 10.1021/jacs.0c04331. 32602703 PMC7368998

[ref32] Liu M.; Wang J.; Chang Y.; Zhang Q.; Chang D.; Hui C. Y.; Brennan J. D.; Li Y. In Vitro Selection of a DNA Aptamer Targeting Degraded Protein Fragments for Biosensing. Angew. Chem., Int. Ed. Engl. 2020, 59 (20), 7706–7710. 10.1002/anie.202000025. 32155319

[ref33] Yao C.; Ou J.; Tang J.; Yang D. DNA Supramolecular Assembly on Micro/Nanointerfaces for Bioanalysis. Acc. Chem. Res. 2022, 55 (15), 2043–2054. 10.1021/acs.accounts.2c00170. 35839123

[ref34] Yao C.; Zhang R.; Tang J.; Yang D. Rolling circle amplification (RCA)-based DNA hydrogel. Nat. Protoc 2021, 16 (12), 5460–5483. 10.1038/s41596-021-00621-2. 34716450

[ref35] Zanoli L. M.; Spoto G. Isothermal amplification methods for the detection of nucleic acids in microfluidic devices. Biosensors (Basel) 2013, 3 (1), 18–43. 10.3390/bios3010018. 25587397 PMC4263587

[ref36] Liao D. F.; Cao C.; Ying Y. L.; Long Y. T. A General Strategy of Aerolysin Nanopore Detection for Oligonucleotides with the Secondary Structure. Small 2018, 14 (18), 1704520 10.1002/smll.201704520. 29603609

[ref37] Wanunu M. Nanopores: A journey towards DNA sequencing. Phys. Life Rev. 2012, 9 (2), 125–158. 10.1016/j.plrev.2012.05.010. 22658507 PMC3780799

[ref38] Soengas M. S.; Gutierrez C.; Salas M. Helix-destabilizing activity of phi 29 single-stranded DNA binding protein: effect on the elongation rate during strand displacement DNA replication. J. Mol. Biol. 1995, 253 (4), 517–529. 10.1006/jmbi.1995.0570. 7473731

[ref39] Ali M. M.; Li F.; Zhang Z.; Zhang K.; Kang D. K.; Ankrum J. A.; Le X. C.; Zhao W. Rolling circle amplification: a versatile tool for chemical biology, materials science and medicine. Chem. Soc. Rev. 2014, 43 (10), 3324–3341. 10.1039/c3cs60439j. 24643375

[ref40] Maglia G.; Restrepo M. R.; Mikhailova E.; Bayley H. Enhanced translocation of single DNA molecules through α-hemolysin nanopores by manipulation of internal charge. Proc. Natl. Acad. Sci. U. S. A. 2008, 105 (50), 19720–19725. 10.1073/pnas.0808296105. 19060213 PMC2604925

[ref41] Wanunu M.; Morrison W.; Rabin Y.; Grosberg A. Y.; Meller A. Electrostatic focusing of unlabelled DNA into nanoscale pores using a salt gradient. Nat. Nanotechnol 2010, 5 (2), 160–165. 10.1038/nnano.2009.379. 20023645 PMC2849735

