# Supplementary material for: Nanopore-Based Fingerprint Immunoassay Based on Rolling Circle Amplification and DNA Fragmentation
Source: ACS Nano. 2023 Mar 6;17(6):5412–20. doi: 10.1021/acsnano.2c09889 (PMC10629239; doi:10.1021/acsnano.2c09889)
Supplement: Supplementary file 1 — nn2c09889_si_001.pdf [file nn2c09889_si_001.pdf]

Supporting Information

Nanopore-Based Fingerprint Immunoassay based on Rolling Circle Amplification and DNA Fragmentation

Xinqi Kang, [†] Connie Wu, [¶a] Mohammad Amin Alibakhshi, [§] Xingyan Liu, [†] Luning Yu, [§] David R. Walt, [¶] and Meni Wanunu[§, ‡]

Departments of [†]Bioengineering, [§]Physics, and [‡]Chemistry and Chemical Biology, Northeastern University, Boston, Massachusetts, 02115, United States

[¶] Department of Pathology, Brigham and Women's Hospital, Harvard Medical School and Wyss Institute for Biologically Inspired Engineering at Harvard University, Boston, Massachusetts, 02115, United States

[a]Present address: University of Michigan Life Sciences Institute, Department of Biomedical Engineering, Ann Arbor, MI, 48109, United States

Table of Contents

Supporting Information 0

I. Template Ligation, optimization of rolling circle amplification, and PAGE characterization of reporter molecules. 1

II. Nanopore measurement of pure DNA hairpins 5

III. Nanopore characterization of DNA reporter molecules 8

IV. Ligation and optimization of methylated templates and nanopore comparison with unmethylated template 10

I. Template Ligation, optimization of rolling circle amplification, and PAGE characterization of reporter molecules.

AluI D6 primer	/5Biosg/AAA AAA AAA AAA AAA CAC TGC TCA CGA CAC GAC TA
AluI D6 template	/5Phos/GTGAGCAGTGAAAAAATTGAAAGCTAACGAAAACGTTAGCTTTCAAAAAAAATAGTCGTGTC
AluI D7 primer	/5Biosg/AAA AAA AAA AAA AAA CAC TGC TCA CGA CAC GAC TA
AluI D7 template	/5Phos/GTGAGCAGTGAAAAAAGTGAAAGCTAACGAAAACGTTAGCTTTCACAAAAAATAGTCGTGTC
RsaI D1 primer	/5BiosG/AAAAAAAAAAAAAAA GTTGACCACTATGTCTTGTG
RsaI D1 template	/5Phos/GGTCAACAAAAAAACAGGTGTACCGAAAAAACGGTACACCTGTAAAAAACACAAGACATAGT
RsaI D2 primer	/5BiosG/AAAAAAAAAAAAAAA GTTGACCACTATGTCTTGTG
RsaI D2 template	/5Phos/GGTCAACAAAAAAGTGGTACCAAGACAAAAGTCTTGGTACCACAAAAAACACAAGACATAGT
AluI D6 primer	/5Biosg/AAA AAA AAA AAA AAA CAC TGC TCA CGA CAC GAC TA
Methylated AluI D6 template	/5Phos/GTG AGC AGT GAA AAA ATT GAA AG/iMe-dC/ TAA CGA AAA CGT TAG /iMe-dC/TT TCA AAA AAA ATA GTC GTG TC

Table S1 Sequences of primers and templates used in this study.

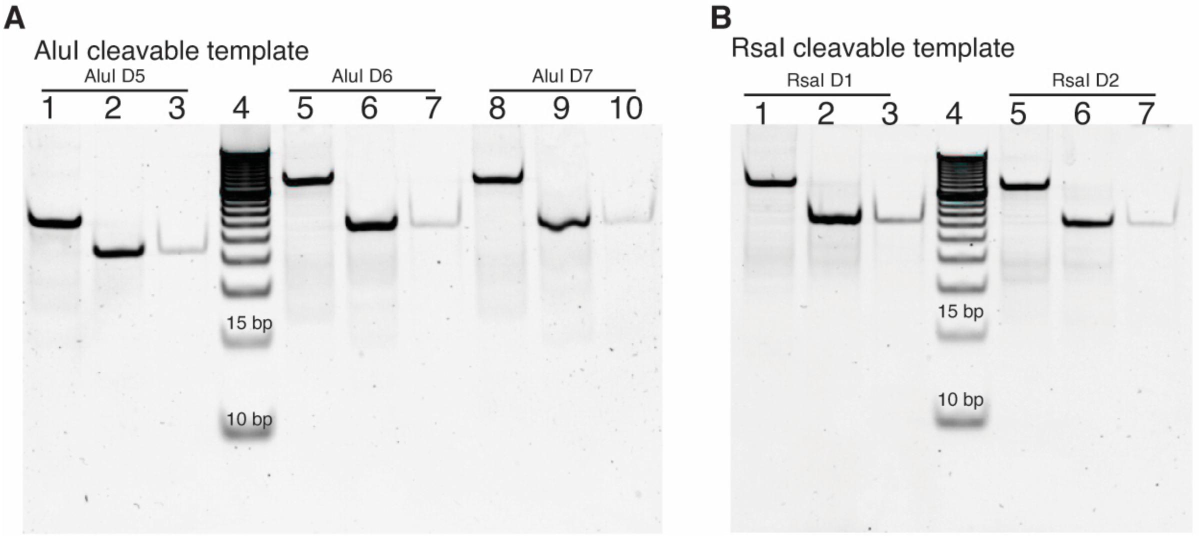

Figure S1. Native PAGE characterization of template ligation. (A) AluI cleavable template ligation. Lane 1-3: linear, circularized AluI D5 template and the Exonuclease I and Exonuclease III treatment of circularized AluI D5 templates. Lane 4: O'RangeRuler 5 bp DNA Ladder. Lane 5-7: linear, circularized AluI D6 template and the Exonuclease I and Exonuclease III treatment of circularized AluI D6 templates. Lane 8-10: linear, circularized AluI D7 template and the Exonuclease I and Exonuclease III treatment of circularized AluI D7 templates. (B) RsaI cleavable template ligation. Lane 1-3: linear, circularized RsaI D1 template and the Exonuclease I and Exonuclease III treatment of circularized RsaI D1 templates. Lane 4: O'RangeRuler 5 bp DNA Ladder. Lane 5-7: linear, circularized RsaI D2 template and the Exonuclease I and Exonuclease III treatment of circularized RsaI D2 templates. Exonuclease I and Exonuclease III treatment is to confirm successful ligation. Because Exonuclease I and Exonuclease III act upon linear ssDNA and dsDNA respectively, while leaving circularized

DNA intact. The gel was run with 20% polyacrylamide and stained with Gelred. The image was visualized with a Biorad system. All circularized DNA samples show a single band which indicates a high ligation efficiency.

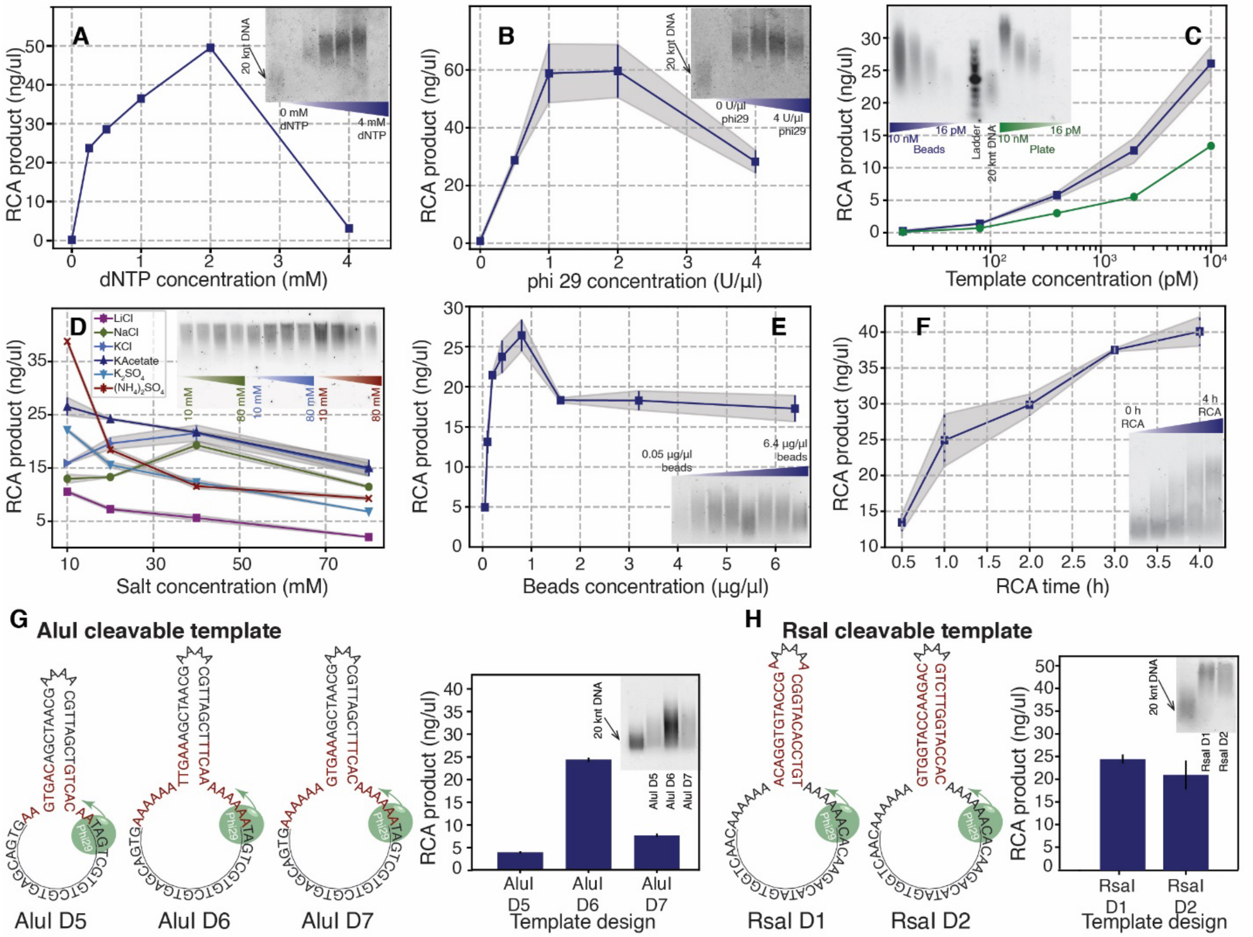

Figure S2. Optimization of the rolling circle amplification reaction. RCA product concentration (ng/μL) as a function of (A) dNTP concentration, (B) phi29 activity, (C) different surfaces for immobilizing primer-template hybrid, (D) salt type and salt concentration, (E) magnetic beads concentration, (F) reaction time. (G) Three DNA templates were designed to produce RCA products that could be cleaved by restriction enzyme AluI (G) or RsaI (H). The three AluI cleavable templates share the same primer sequence (labeled with an underline). The difference in the sequence is labeled in red and the same sequence is labeled in black. Same color coding is also used in RsaI cleavable template. Inset shows the 0.3% agarose gel electrophoresis result of formamide denatured RCA product and 20 kbp Nolimit DNA as a reference. All primer-template hybrids were immobilized on streptavidin-coated magnetic beads except for (A) and (B) in which the primer-template hybrid was not immobilized on any surface, and (C) in which primer-template hybrid was immobilized on either streptavidin-coated magnetic beads or streptavidin-coated plates. The reaction time is 1 hour except for 2 hours for (D). All reaction conditions were 10 mM $(NH_4)_2SO_4$, 10 mM $MgCl_2$, 50 mM Tris-HCl pH 7.5, 4 mM DTT, 0.05% Tween-20, 1 mM dNTP, 2 U/μL phi29 polymerase, 1 μg/μL 2.8 μm magnetic beads, 37 °C and 10 nM initial template concentration unless stated otherwise.

Optimization nanopore characterization of reporter molecules

We optimized the RCA reaction performance as a function of dNTP concentration, phi29 activity, surface (magnetic beads versus ELISA plates), salt type and concentration, magnetic bead concentration, and RCA reaction time. Circular template AluI design 6

(AluI D6) was used in all optimization experiments. After the reaction was completed, we used formamide to disrupt streptavidin-biotin binding to release immobilized nucleic acids from the surface as well as denature the secondary structure of the RCA product. The total mass concentration of the RCA product was quantified using Qubit single strand staining dye, and the RCA product length was visualized using 0.3% agarose gel electrophoresis.

As shown in Figure S2A, the RCA product mass concentration increased and then decreased with dNTP concentration. At low concentration of dNTP, both RCA product mass and RCA product length, as shown in the agarose gel in the inset, are lower. This could be due to the long waiting time for the next dNTP to diffuse to the phi29 polymerase amplification site. On the other hand, at high dNTP concentration, dNTP will chelate magnesium (Mg^{2+}) which then influences polymerase activity and results in a low RCA product mass. When increasing phi29 polymerase activity, we observed an increase and then decrease in RCA product mass (Figure S2B).[2] The increase in product mass at low phi29 polymerase activity could be attributed to increasing amounts of enzyme involved in the reaction, while the decrease at high phi29 polymerase activity might be due to increased glycerol concentration (co-solvent in the phi29 stock solution).

To quantify the RCA product mass and length on surfaces, the primer and template conjugate were immobilized on streptavidin-coated surfaces, and 95% formamide, 10mM EDTA, pH 8.2, 90 °C 30 min incubation was performed to break the streptavidin-biotin bound after the reaction was finished. Generally, plates have a lower surface-to-volume ratio and phi29 polymerases need to diffuse and bind to primer-template hybrids which are located at the bottom of the ELISA well. Both factors lead to a lower binding of phi29

polymerases to primer-template hybrids. Thus, comparing the two different surfaces, magnetic beads showed higher RCA product mass when the initial circular template concentration was the same (Figure S2C), however, the average RCA product length is shorter than in the bead reaction (Figure S2C inset).

Salt is critical for bioassays. We examined eight different salts, and our result shows that 10 mM NH_3SO_4 provides the highest RCA rate and LiCl is the lowest (Figure S2D). When comparing LiCl, NaCl, and KCl, which are salts with different cations and the same anion, we observe that K^+ gives the highest enzyme performance. Compared to KCl, K_2SO_4, and KCH_3CO_2, SO_4^{2-} and $CH_3CO_2^-$ all perform better$^-$, in accordance with the Hofmeister series of ion-specific effects on proteins and their biological functions.[3, 4] Based on the Hofmeister series, the order of cations being hydrated in the buffer is $K^+ > Na^+ > Li^+$.[3, 4] Strongly hydrated ions tend to have higher surface tension, lower hydrocarbon solubility, and stabilize native protein structure.[3] Compared with SO_4^{2-} and acetate$^-$, Cl^- is poorly hydrated and tends to facilitate protein unfolding and denaturation.[4] An interesting observation is that the difference in salt type and concentration does not affect RCA product length (Figure S2D inset). The RCA product mass shows an increase and then decrease as a function of beads concentration (Figure S2E), which might be caused by the absorption of phi29 polymerase and DNA oligos to the surface of the beads. And finally, the RCA product mass and length show an increase as a function of time as expected (Figure S2F).[1]

The circular templates include a DNA hairpin structure that requires unfolding by phi29 polymerase during RCA. Thus, the sequence and the length of DNA hairpin structure can potentially influence the speed of phi29 polymerase. To examine this hypothesis, and to

optimize our final DNA reporter molecule yield, we design several circular templates with the same primer sequence. The first set of circular templates is cleaved by the AluI restriction enzyme (Figure S2G). The primer binding sequence is underlines, and the difference in sequences is marked in red. Considering the steric hindrance, we inserted several poly adenosines (poly-A) between the primer binding sequence and the DNA hairpin secondary structure. While the remaining lengths of DNA hairpins are the same 13 bps, AluI design 5 (AluI D5) has poly-A_2, AluI design 6 (AluI D6) and AluI design 7 (AluI D7) have poly-A_6 between the primer binding sequence and the DNA hairpins.The first base pair that phi29 polymerase needs to unfold in DNA hairpin structures is the AT pair for AluI D6, and the GC pair for AluI D5 and AluI D7. The results show that AluI D6 has both the highest RCA product mass and length followed by AluI D7 and AluI D5. Thus, we reason that the overall steric hindrance, the kinetics of phi29 unfolding first base pair, and the overall GC content will influence the efficiency of amplification. The second set of circular templates os cleaved by restriction enzyme RsaI (Figure S2H). For these two RsaI cleavable templates, the length of DNA hairpin secondary structures is 12 bp for RsaI D1 and 13 bp for RsaI D2. Poly-A_6 has been put in between the primer binding sequence and the DNA hairpin secondary structure, and the GC content of the DNA hairpin stem is almost the same, with RsaI D2 slightly lower. We did not observe a strong RCA product mass and length difference between these two templates. Overall, this result implies that by fine-tuning the steric hindrance, hairpin stem length, and GC content, phi29 performance can be optimized.

II. 9 Nanopore measurement of pure DNA hairpins

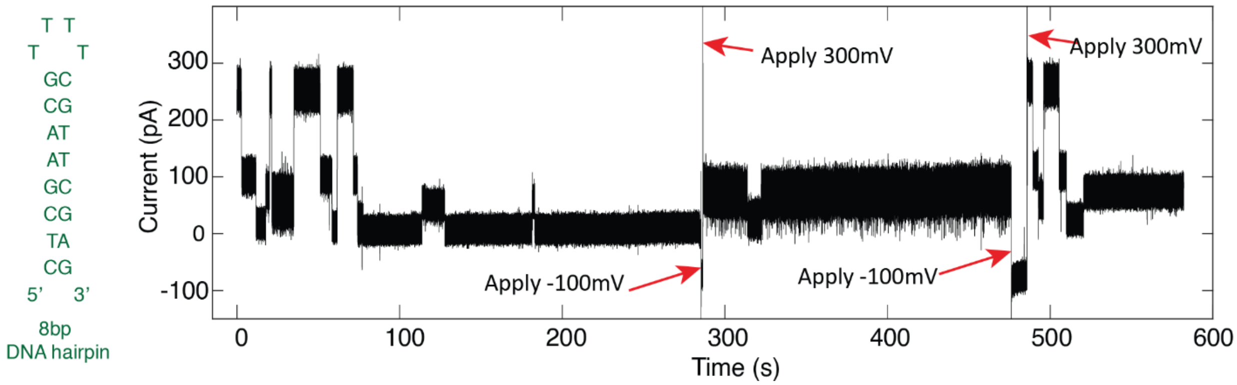

Figure S3. Sequence, structure, current trace of 8bp DNA hairpin. Multiple steps were observed in a single event with first level blockade around 58% blockade. And the largest blockade level in a single event could up to 96 %, Some events have more than 100 s dwell time and only leave alpha-hemolysin upon reversing the voltage polarity. All measurements were performed with 1M KCl, 20 mM Tris, pH 7.6, at 300 mV applied bias, and the current signal was lowpass filtered at 10 kHz.

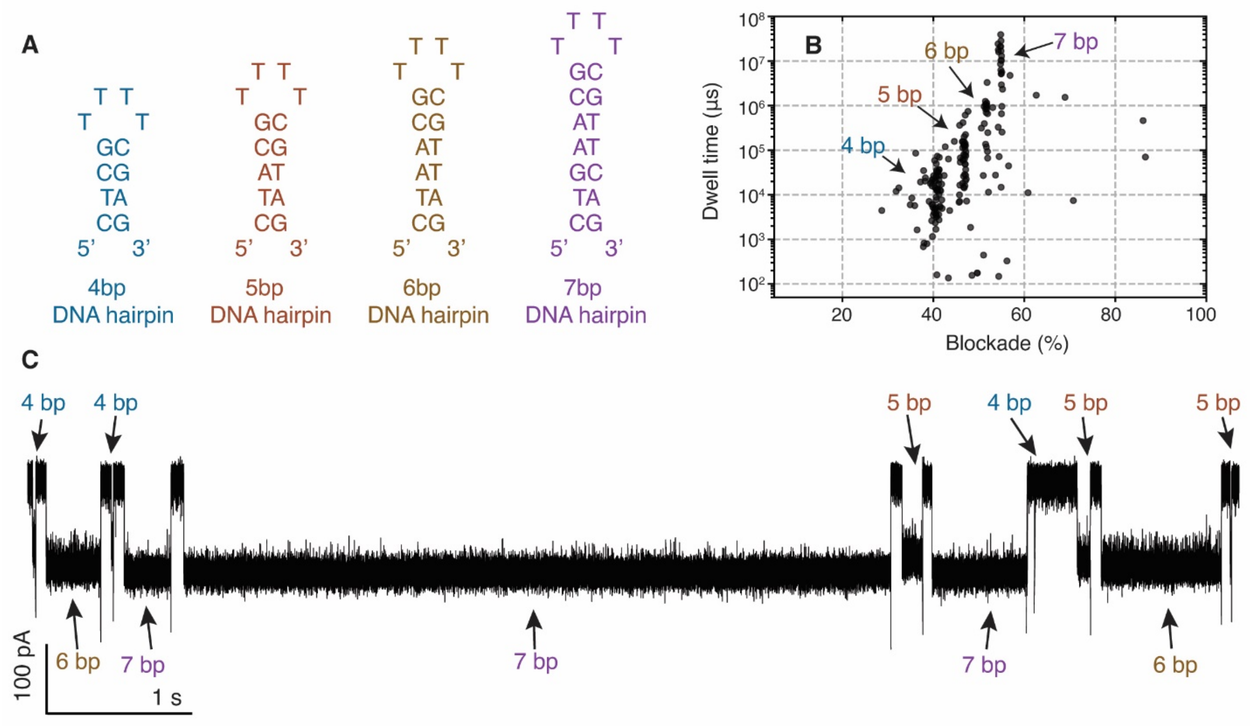

Figure S4. Sequence, structure, dwell time versus blockade scatter plot and the current trace of DNA hairpins. (A) 4 bp, 5 bp, 6 bp, and 7 bp DNA hairpin sequences and the predicted secondary structure. (B) Scatter plot of dwell time versus fractional blockade for the four-hairpin mixture. (C) Current trace of the four-hairpin mixture translocation through alpha-hemolysin pore. All measurements were performed with 1M KCl, 20 mM Tris, pH 7.6, at 250 mV applied bias, and each hairpin concentration was 250 nM. The current signal was lowpass filtered at 10 kHz.

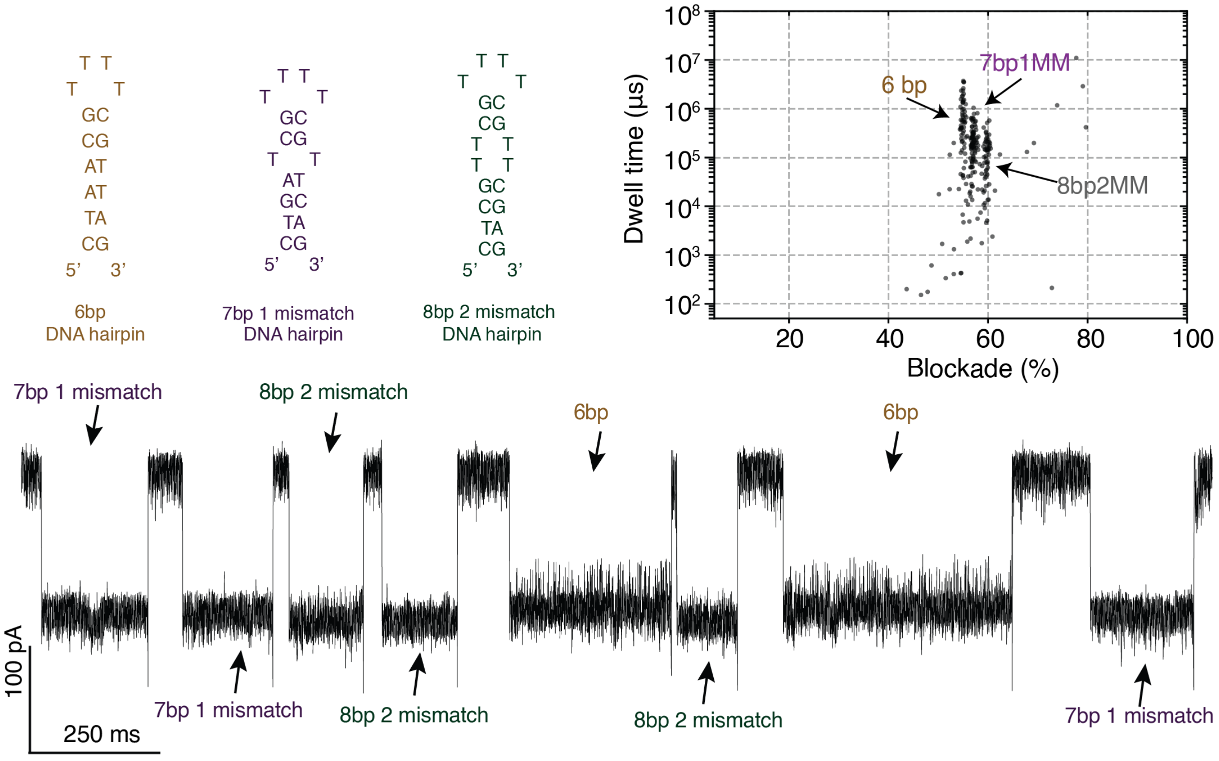

Figure S5. Sequence, structure, current trance, and dwell time versus blockade scatter plot of DNA hairpin. Sequences, predicted structures, dwell time versus fractional blockade scatter plot, and a current trace of 6 bp, 7 bp 1 mismatch, and 8 bp 2 mismatch hairpin. Mismatches in the hairpin reduce the unfolding energy resulting in reduced dwell time. All measurements were performed with 1M KCl, 20 mM Tris, pH 7.6, at 300 mV applied bias, and the current signal was lowpass filtered at 10 kHz.

III. Nanopore characterization of DNA reporter molecules

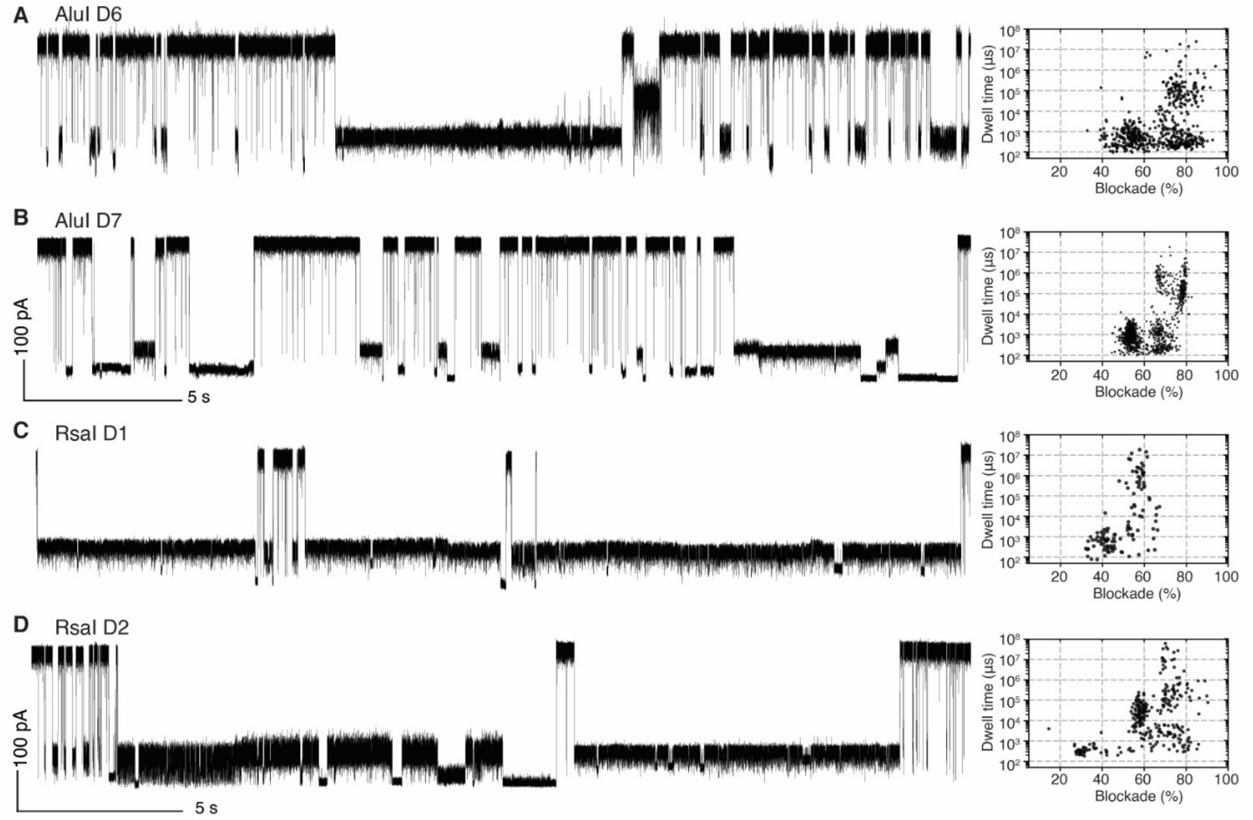

Figure S6. Continues current trace and the scatters plot of reporter molecules. Ionic current trace obtained from reporter molecules of AluI D6 (A), AluI D7 (B), RsaI D1 (C), and RsaI D2 (D). A corresponding scatters plot is shown on the left. All measurements were performed with 1M GdmCl, 1M KCl, 20 mM Tris, pH 7.6, at 250 mV applied bias, and the current signal was lowpass filtered at 10 kHz.

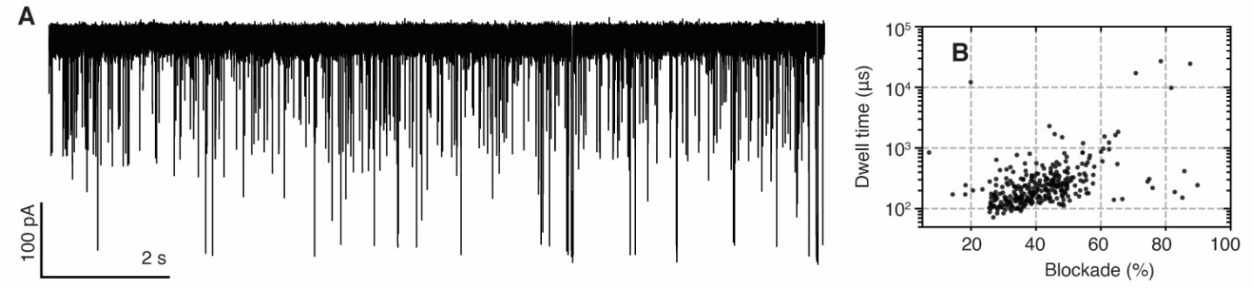

Figure S7. Ionic current trace and scatter plot of AluI D6 reporter molecules heat incubated in 1M GdmCl. AluI D6 reporter molecules with an initial circular template concentration of 10 nM were incubated at 37 ℃ with 1 M GdmCl, 1 M KCl, 50mM Tris, pH 7.6 for 1 h. Then nanopore measurement was performed with the same buffer, at 250 mV applied bias, lowpass filtered at 10 kHz. The current trace (A), and scatter plot (B) were obtained.

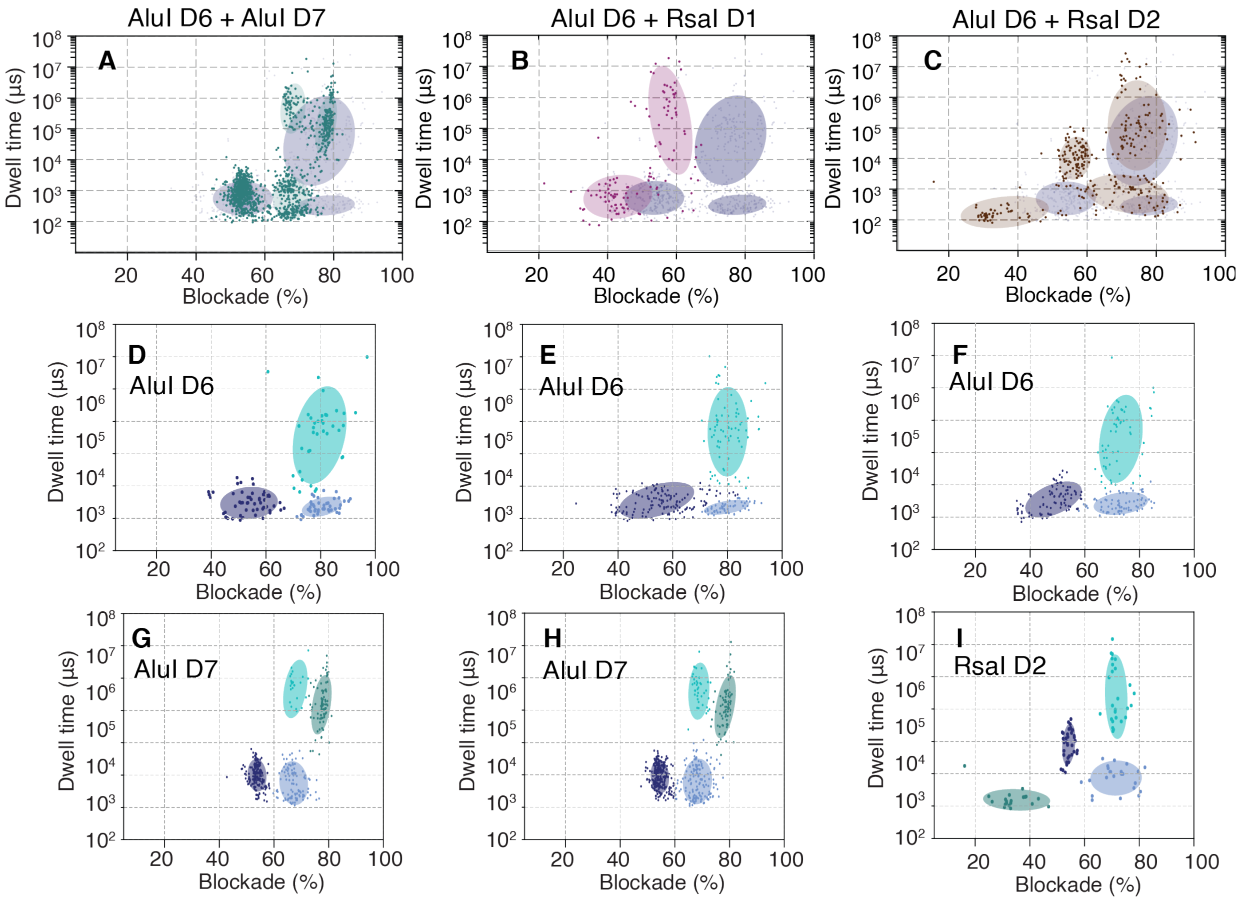

Figure S8. Distinctive scatter plots and reproducibility between different pores. Overlayed scatter plots of AluI D7 (A), RsaI D1 (B), and RsaI D2 (C) over AluI D6. The AluI D6 is shown as blue and AluI D7, RsaI D1, and RsaI D2 are shown in green, purple, and brown, respectively. Scatter plot obtained from multiple independent experiment of AluI D6 reporter molecules (D-F), AluI D7 reporter molecules (G-H), RsaI D2 reporter molecules (I). All measurements were performed with 1M GdmCl, 1M KCl, 20 mM Tris, pH 7.6, at 250 mV applied bias, and the current signal was lowpass filtered at 10 kHz.

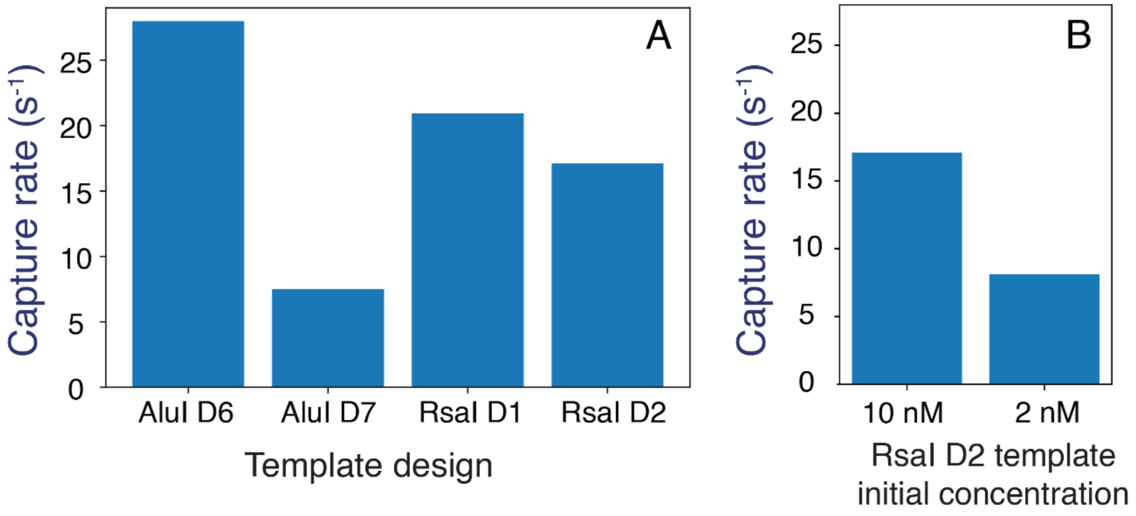

Figure S9. Capture rate comparison of different templates. (A) Comparison of different circular DNA templates at initial concentration of 10 nM. (B) Capture rate of RsaI D2 at different initial concentration.s Nanopore experiments were performed in 1 M GdmCl, 1 M KCl, 50 mM Tris, pH 7.6, at 250 mV applied bias. The current signal was lowpass-filtered at 10 kHz.

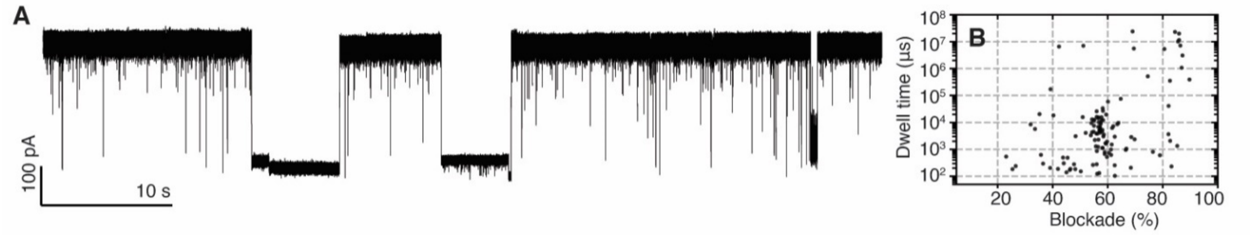

Figure S10. IFN gamma detection using RsaI D2. Magnetic beads and primer-RsaI D2 template hybrid were conjugated with interferon-gamma capture antibody and detector antibody. After the formation of the sandwich ELISA structure, an RCA reaction was performed, and reporter molecules were released by the RsaI enzyme. Nanopore measurements were conducted in 1 M GdmCl, 1 M KCl, 50 mM Tris, pH 7.6 buffer. The current trace (A) and scatter plot of dwell time versus fractional blockade (B) were obtained.

IV. Ligation, optimization, of methylated templates and nanopore comparison with unmethylated template

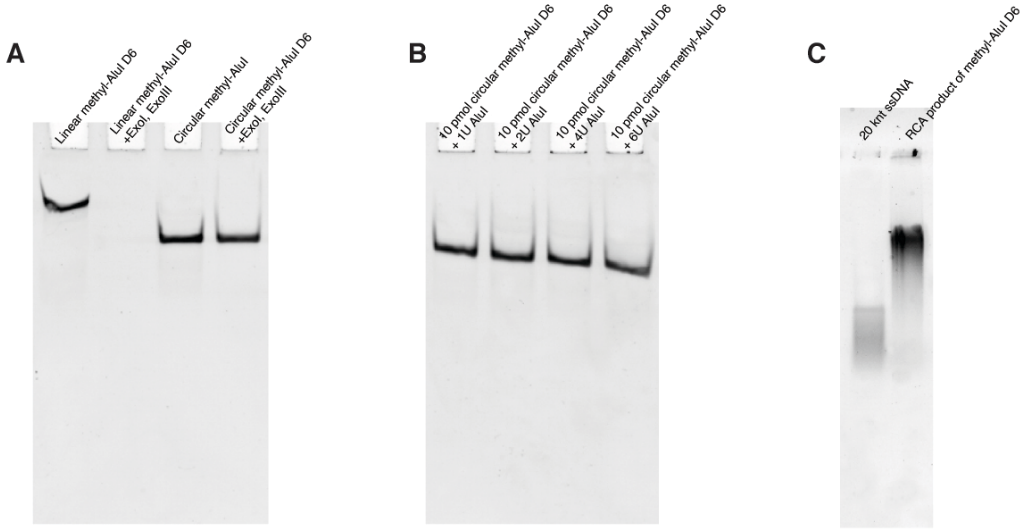

Figure S11. Characterization of methylated AluI D6 template. (A) Circularization of the linear template. The linear and circularized template shows a different migration speed on 20% native PAGE gel. Treatment of linear template with ExoI and ExoIII shows complete digestion while the circularized template is resistant to ExoI and ExoIII. (B) AluI resistant assay of methylated AluI D6. After circularization, the templates were titrated with AluI. 20 % native PAGE and Gelred was used for visualization. The distorted band is due to the high concentration of AluI protein in the sample. (C) RCA reaction with methyl-AluI D6. The reaction condition was 10 mM $(NH_4)_2SO4$, 10 mM $MgCl_2$, 50 mM Tris-HCl pH 7.5, 4 mM DTT, 1 mM dNTP, 2 U/µL phi29 polymerase, 1 µg/µL 2.8 µm magnetic beads, 37 ℃ for 1h. After the reaction, the sample and the 20 kbp Nolimit DNA were incubated in 95% formamide, 10mM EDTA, pH 8.2, at 90 ℃ for 15 min. And 0.3 % agarose gel was used to compare the relative length.

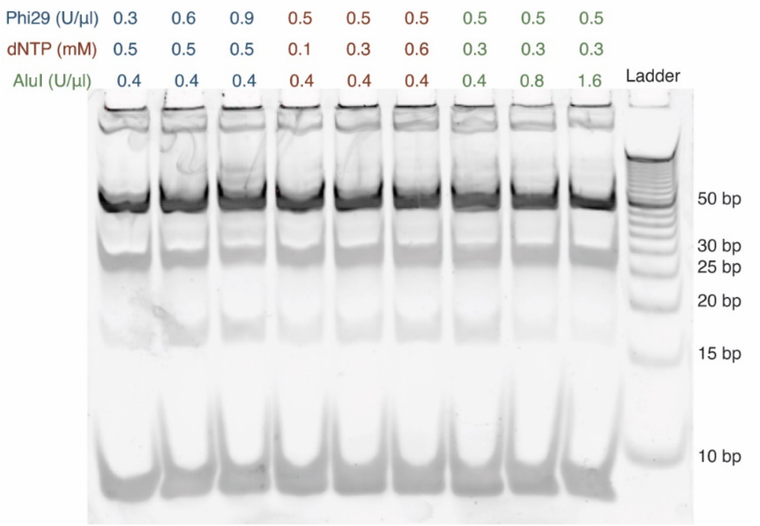

Figure S12. Influence of reaction parameters for INCA. To optimize the yield of as well as understand the possible structure and size reporter molecules produced via INCA. We changed the dNTP concentration, phi29 unit, AluI unit and compared the results. No significant difference was observed in the range of parament we choose. The reaction temperature and time were 37 ℃, and 1 h. the primer-template hybrid was immobilized on streptavidin beads.

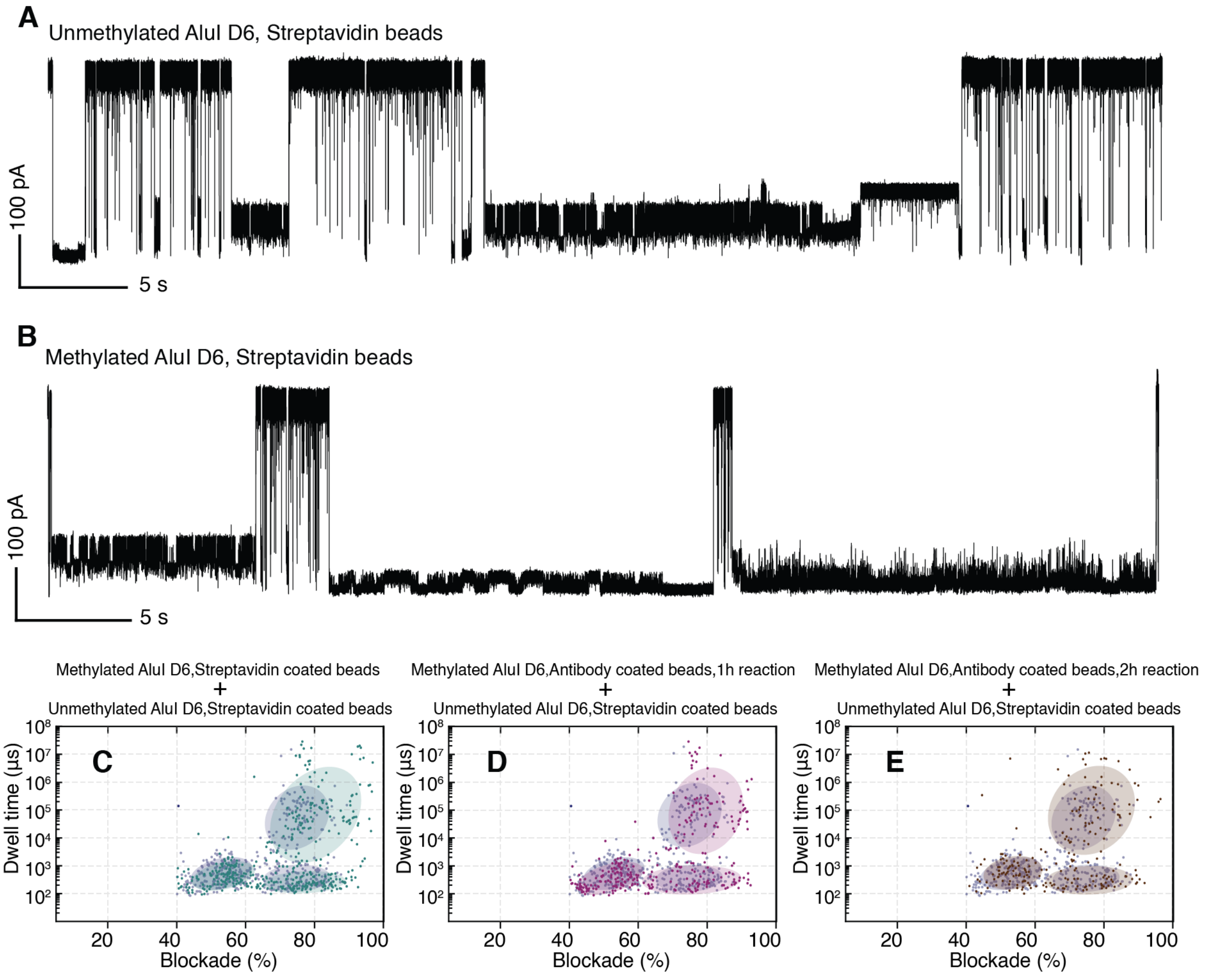

Figure S13. Comparison of unmethylated and methylated AluI D6. Current trace of methylated (A) and unmethylated AluI D6 (B). Both initial template concentrations were 400 pM and immobilized on 1 µg/µl 2.8 µm magnetic beads. Unmethylated AluI D6 was RCA amplified first and then cleavage by AluI while methylated AluI D6 was done via INCA. Overlayed scatter plots of methylated AluI D6 on streptavidin coated beads (C), methylated AluI D6 on antibody coated beads (D) (E) over unmethylated AluI D6. The unmethylated AluI D6 was shown as blue. After reporter molecule generation, nanopore measurements were performed in 1 M GdmCl, 1 M KCl, 50 mM Tris, pH 7.6, 250 mV applied bias, lowpass filtered at 10 kHz.

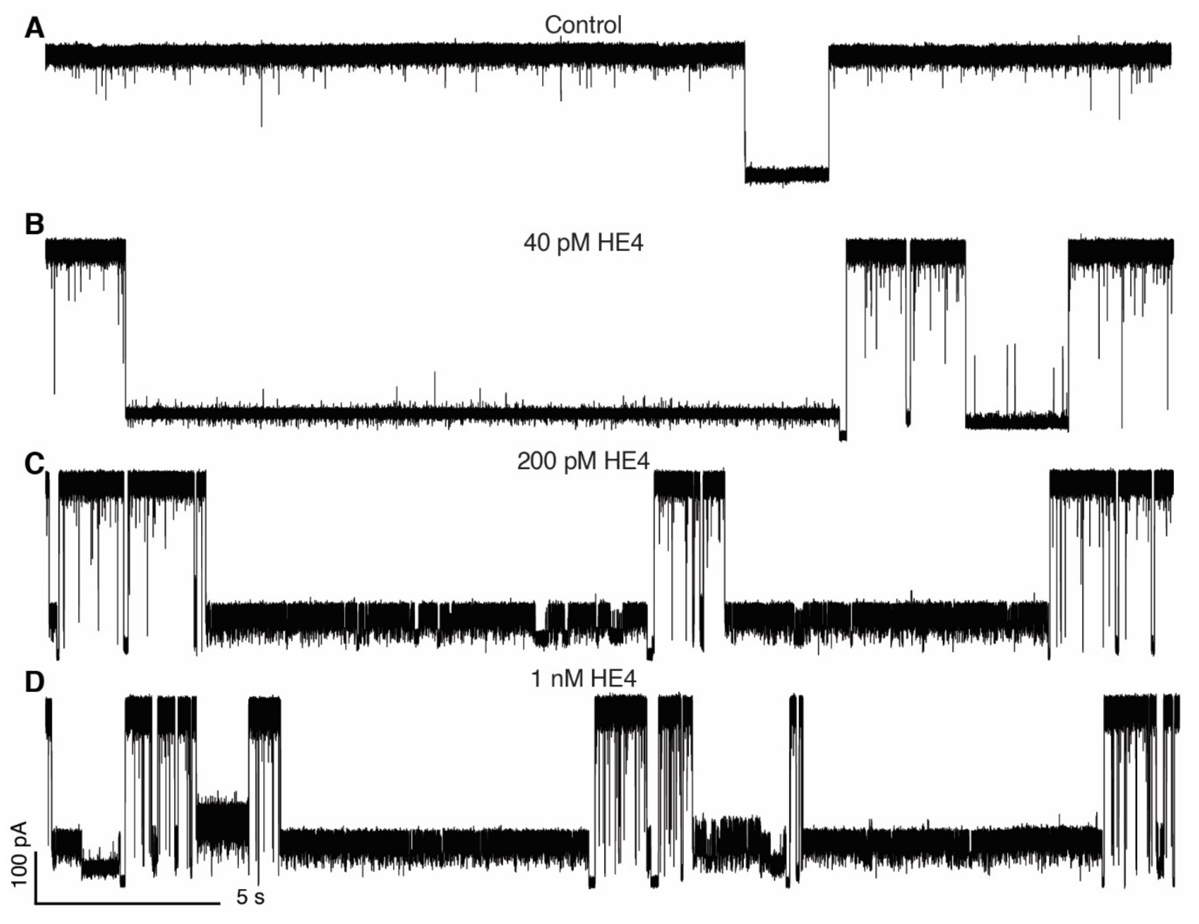

Figure S14. HE4 quantification using methylated circular DNA template and 1 h reaction time. Representative current traces recorded from blank control (A), 40 pM (B), 200 pM (C), and 1 nM HE4 (D). Experiments were performed in 1M GdmCl, 1M KCl, 50 mM Tris, pH 7.6, at 250 mV applied bias. The current signal was lowpass filtered at 10 kHz.

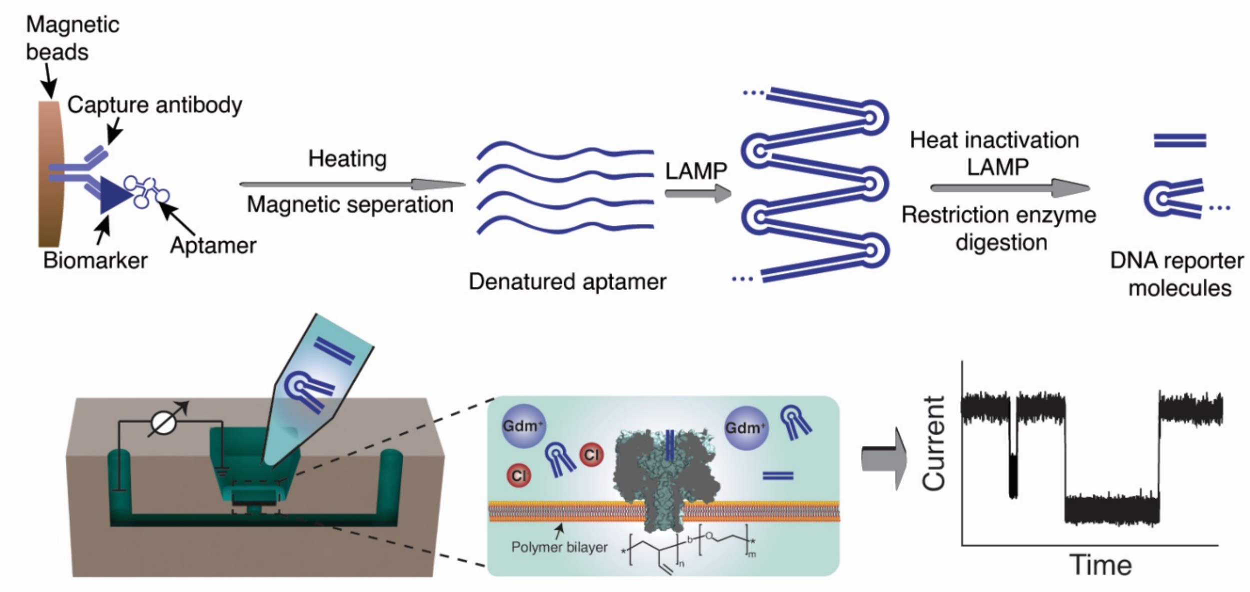

Figure S15. Schematic of LAMP assay for nanopore amplification of reporter molecules. After the formation of sandwich ELISA, a heat release of aptamer molecules, loop-mediated isothermal amplification, and 80 °C heat inactivation of reaction, we will have long dsDNA products. And by using restriction enzymes to digest long dsDNA products into DNA fragments, which are reporter molecules in this schematic, we could use alpha-hemolysin pore for biomarker quantification.

(1) Lin, L.; Li, B.; Han, X.; Zhang, F.; Zhang, X.; Linhardt, R. J. A rolling circle amplification based platform for ultrasensitive detection of heparin. *Analyst* **2021**, *146* (2), 714-720. DOI: 10.1039/d0an02061c. Murakami, T.; Sumaoka, J.; Komiyama, M. Sensitive isothermal detection of nucleic-acid sequence by primer generation-rolling circle amplification. *Nucleic Acids Res* **2009**, *37* (3), e19. DOI: 10.1093/nar/gkn1014.

(2) Li, Y.; Dai, W.; Lv, X.; Deng, Y. Aptamer-based rolling circle amplification coupled with graphene oxide-based fluorescence resonance energy transfer for sensitive detection of cardiac troponin I. *Analytical Methods* **2018**, *10* (15), 1767-1773. DOI: 10.1039/c8ay00309b.

(3) Kang, B.; Tang, H.; Zhao, Z.; Song, S. Hofmeister Series: Insights of Ion Specificity from Amphiphilic Assembly and Interface Property. *ACS Omega* **2020**, *5* (12), 6229-6239. DOI: 10.1021/acsomega.0c00237.

(4) Okur, H. I.; Hladilkova, J.; Rembert, K. B.; Cho, Y.; Heyda, J.; Dzubiella, J.; Cremer, P. S.; Jungwirth, P. Beyond the Hofmeister Series: Ion-Specific Effects on Proteins and Their Biological Functions. *J Phys Chem B* **2017**, *121* (9), 1997-2014. DOI: 10.1021/acs.jpcb.6b10797.
